# Crystal structure of the TreS:Pep2 complex, initiating α-glucan synthesis in the GlgE pathway of mycobacteria

**DOI:** 10.1074/jbc.RA118.004297

**Published:** 2019-03-15

**Authors:** Ali A. Kermani, Rana Roy, Chai Gopalasingam, Klaudia I. Kocurek, Trushar R. Patel, Luke J. Alderwick, Gurdyal S. Besra, Klaus Fütterer

**Affiliations:** From the Institute of Microbiology & Infection, School of Biosciences, University of Birmingham, Edgbaston, Birmingham B15 2TT, United Kingdom

**Keywords:** mycobacteria, analytical ultracentrifugation, X-ray crystallography, trehalose, enzyme catalysis, protein complex, pathogenesis, α-glucan, capsule, immune evasion, maltose kinase, trehalose synthase

## Abstract

A growing body of evidence implicates the mycobacterial capsule, the outermost layer of the mycobacterial cell envelope, in modulation of the host immune response and virulence of mycobacteria. Mycobacteria synthesize the dominant capsule component, α(1→4)-linked glucan, via three interconnected and potentially redundant metabolic pathways. Here, we report the crystal structure of the *Mycobacterium smegmatis* TreS:Pep2 complex, containing trehalose synthase (TreS) and maltokinase (Pep2), which converts trehalose to maltose 1-phosphate as part of the TreS:Pep2–GlgE pathway. The structure, at 3.6 Å resolution, revealed that a diamond-shaped TreS tetramer forms the core of the complex and that pairs of Pep2 monomers bind to opposite apices of the tetramer in a 4 + 4 configuration. However, for the *M. smegmatis* orthologues, results from isothermal titration calorimetry and analytical ultracentrifugation experiments indicated that the prevalent stoichiometry in solution is 4 TreS + 2 Pep2 protomers. The observed discrepancy between the crystallized complex and the behavior in the solution state may be explained by the relatively weak affinity of Pep2 for TreS (*K_d_* 3.5 μm at mildly acidic pH) and crystal packing favoring the 4 + 4 complex. Proximity of the ATP-binding site in Pep2 to the complex interface provides a rational basis for rate enhancement of Pep2 upon binding to TreS, but the complex structure appears to rule out substrate channeling between the active sites of TreS and Pep2. Our findings provide a structural model for the trehalose synthase:maltokinase complex in *M. smegmatis* that offers critical insights into capsule assembly.

## Introduction

Through co-evolution with its host over several millennia, *Mycobacterium tuberculosis,* the organism causing tuberculosis (TB),^5^ has developed mechanisms to partially blunt the human immune response. A key role in this finely tuned host–pathogen interaction is played by various components of the complex mycobacterial cell wall, notably lipoarabinomannan and phosphatidylinositol-anchored mannan (1). Compared with these immunomodulatory cell wall components, the mycobacterial capsule has received relatively little attention. Yet, increasing evidence points to a role of the capsule in immune modulation and virulence. For instance, destruction of the capsule by sonication promotes phagocytosis *ex vivo* (2); capsular polysaccharides bind with high selectivity to DC-SIGN, a key receptor of the innate immune system on dendritic cells (3); deletion of the *glgA* gene, which encodes a component of the “classical” pathway of α-glucan synthesis, perturbs persistence of bacilli in a mouse infection model for TB (4); and phagocytosis of nonopsonized bacilli by neutrophils appears to be mediated by capsular α-glucan (5). Finally, double knockout mutants deleting ADP-glucose pyrophosphorylase–encoding *glgC* and trehalose synthase–encoding *treS* led to severe capsule-deficiency and significantly reduced virulence of *M. tuberculosis* in a mouse infection model (6), underscoring the significance of the capsule in TB infections.

**Figure 1.**
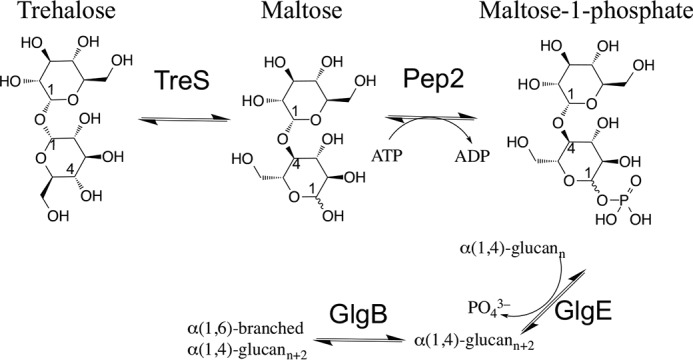
**Schematic diagram of the GlgE pathway of mycobacterial α-glucan synthesis.** Conversion of trehalose to maltose 1-phosphate proceeds through isomerization (TreS) and phosphorylation (Pep2) of the disaccharide (generated using ChemBioDraw).

How the capsule is synthesized and assembled is only partially understood. The capsule material is predominantly made of α-glucan (80–90%), alongside other key components, including arabinomannan (10–20%) and proteins of the ESX-1 secretion system (7). Capsular α-glucan is a polymer of α(1→4)-linked glucose units with α(1→6)-branching and thus is structurally closely related to intracellular glycogen (8, 9). To date, three mycobacterial pathways have been implicated in α-glucan (and/or glycogen) synthesis: the classical GlgC–GlgA pathway (also present in many other bacteria), the Rv3032 pathway (which leads to methylglucose lipopolysaccharide (10)), and the recently identified TreS-Pep2-GlgE (Fig. 1) (9, 11, 12). These pathways share either common nodes or are linked by synthetic lethal interactions (9, 11, 13), conferring an intriguing level of metabolic redundancy. Recent evidence has shown that simultaneous knockout of the classical pathway (GlgC–GlgA) and the GlgE pathway by deleting *glgC* and *treS* abolishes capsule formation and significantly reduces virulence, whereas single knockouts of these two genes partially preserve α-glucan synthesis and have differential effects on α-glucan content in the capsule and the cytosol (6).

In a previous study, we had described the crystal structure of trehalose synthase (TreS) of *M. tuberculosis* (14), which constitutes the entry point to the four-step GlgE pathway of α-glucan synthesis (Fig. 1). In the same study, we showed that TreS, which interconverts trehalose and maltose (15), forms a noncovalent complex *in vitro* and *in vivo* with maltokinase (Pep2), the second enzyme in the GlgE pathway. Pep2 catalyzes phosphate transfer from ATP to maltose (16), yielding maltose 1-phosphate, which is subsequently polymerized by GlgE into α(1→4)-linked glucan (12). In a fourth reaction step, GlgB introduces α(1→6)-branching of α(1→4)-linked glucan chains (17). Contrary to GlgE or GlgB, neither TreS nor Pep2 are essential enzymes in *Mycobacterium smegmatis*, although transposon mutagenesis suggested the essentiality of *M. tuberculosis* Pep2 (18). However, they have been shown to form synthetic lethal interactions with glycosyltransferase Rv3032 (which synthesizes linear α-glucan from UDP-glucose) and thus appear to occupy an important metabolic node (11).

The TreS:Pep2 complex of *M. tuberculosis* contains a tetramer of TreS and four copies of Pep2, with an overall mass of 490 kDa (14). TreS and Pep2 can work independently, but we observed that when *M. tuberculosis* TreS is added to the *M. tuberculosis* Pep2-catalyzed phosphorylation of maltose, Pep2 activity increased markedly (14). This raised the question by what mechanism TreS is able to affect the catalytic activity of Pep2 and/or whether substrate channeling may be part of the TreS:Pep2 interaction. To help address these questions, we undertook the crystallization of the TreS:Pep2 complex. Crystals of the *M. tuberculosis* complex failed to provide useful diffraction patterns, whereas diffraction data could be obtained with crystals of the orthologues from *M. smegmatis*, diffracting to a resolution of 3.6 Å.

## Results

### X-ray crystal structure of the M. smegmatis TreS:Pep2 complex

**Figure 2.**
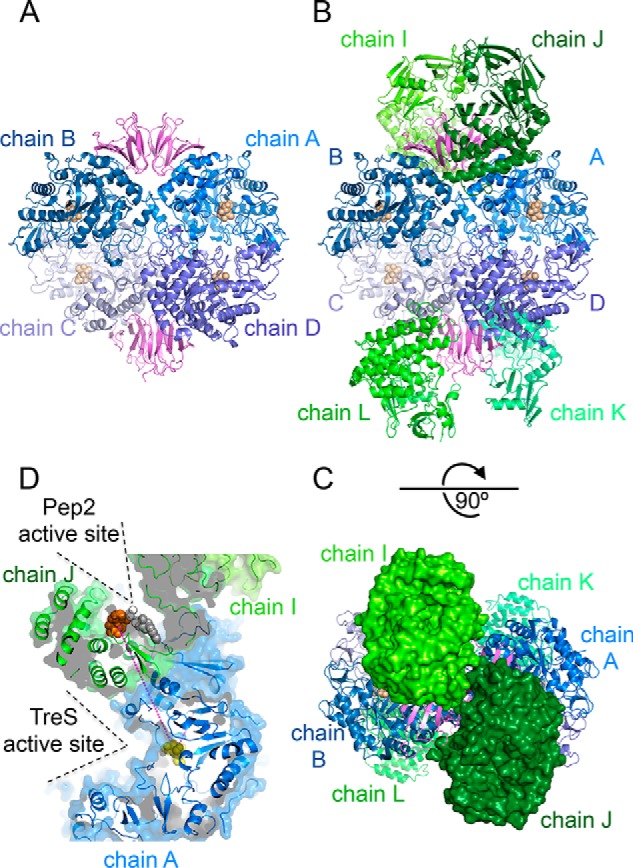
**Architecture of the *M. smegmatis* TreS:Pep2 complex.** *A,* view of the TreS tetramer, illustrating juxtaposition of the C-terminal β-sandwich domains (*hues of magenta*) at the apices of the tetramer. The tetramer shown is from the first of two complex copies in the asymmetric unit (complex 1) with chain identifiers *A–D. B,* assembly of the TreS:Pep2 complex in the orientation of *A*, with TreS and Pep2 subunits shown in *blue* and *green ribbons*, respectively. *Spheres* indicate the location of the active sites, as derived from the superposition with substrate-bound homologous structures. *Capital letters* designate the chain identifiers in complex 1. *C,* top view of the assembly shown in *B*, with Pep2 subunits rendered as molecular surfaces. The rotation relative to *B* is indicated. *D,* illustration of the relative spatial positions of the active sites of TreS and Pep2. The *line in magenta* shows the linear distance (53 Å) between the disaccharide-binding sites, and the openings of the actives sites to solvent are marked.

The complex of *M. smegmatis* (*Msm*) TreS:Pep2 crystallized readily, but crystals of appropriate quality only grew over a narrow pH range (pH 6.7 to 7.0). The structure was phased by molecular replacement and refined to a resolution of 3.6 Å (Fig. 2 and Table S1). Given the limited resolution, noncrystallographic symmetry (NCS) restraints were applied. The core of the assembly is the previously reported TreS tetramer (Fig. 2*A*) (14). In the diamond-shaped TreS tetramer, the β-sandwich domains of the TreS subunits are paired up and constitute the “top” (chains A and B) and “bottom” apex (chains C and D) of the diamond (Fig. 2*A*). In the hetero-octameric TreS:Pep2 complex, pairs of Pep2 monomers straddle across the β-sandwich domains of the TreS tetramer in a clamp-like fashion (Fig. 2*B*). We refer to this assembly as the 4 + 4 complex, which measures ∼175 Å in its long dimension, ∼125 Å across, and ∼90 Å in depth. Pep2 appears to dimerize on the TreS tetramer, but the contact surface between the Pep2 monomers is small compared with the contact surface with TreS (see Fig. 2*C*), suggesting they bind independently as monomeric copies.

The asymmetric unit (ASU) of the crystal lattice contains two copies of the TreS:Pep2 complex (complexes 1 and 2), which display differences in terms of subunit orientation and level of disorder. For instance, when superimposing the TreS tetramers of complexes 1 (chains A–D) and 2 (chains E–H) onto the structure of stand-alone *M. smegmatis* TreS (PDB code 3ZO9 (19)), the average displacement (RMSD) of Cα atoms is very small for complex 1 (0.45 Å for 2243 aligned Cα atoms) but considerably larger for complex 2 (RMSD of 3.4 Å, 2175 aligned Cα atoms). The origins of this mismatch are rotations of the TreS protomers relative to each other, evident by altered gaps between the subunits' molecular surfaces when oriented according the alignment described above and by changes of the distances between the substrate-binding sites of up to about 6 Å (Fig. S1).

The backbone of Pep2 shows various levels of disorder across the eight protomers in the ASU. In complex 1 (Pep2 chains I–L, Fig. 2*B*), the backbone of Pep2 could be traced over 436 of the 441 residues for three subunits (chains I, J, and L), but residues 1–86 are largely disordered in the fourth subunit (chain K). In complex 2 (Pep2 chains M–P), only one copy of Pep2 (chain M) could be traced fully, whereas residues 1–194 were disordered in chains N, O, and P.

### Structure of Pep2

The structural model of *Msm* Pep2 in the present complex structure is completely defined by electron density bar two residues of the N-terminal sequence and 4–5 residues at the C terminus. Despite the moderate resolution, the density displays sufficient detail to dock the Pep2 amino acid sequence onto the backbone structure, aided in part by the recent structures of *M. tuberculosis* (*Mtb*) and *Mycobacterium vanbaalenii* (*Mvb*) Pep2 (20, 21) (Fig. S2). However, side chains were omitted from the model, where corresponding density was missing.

**Figure 3.**
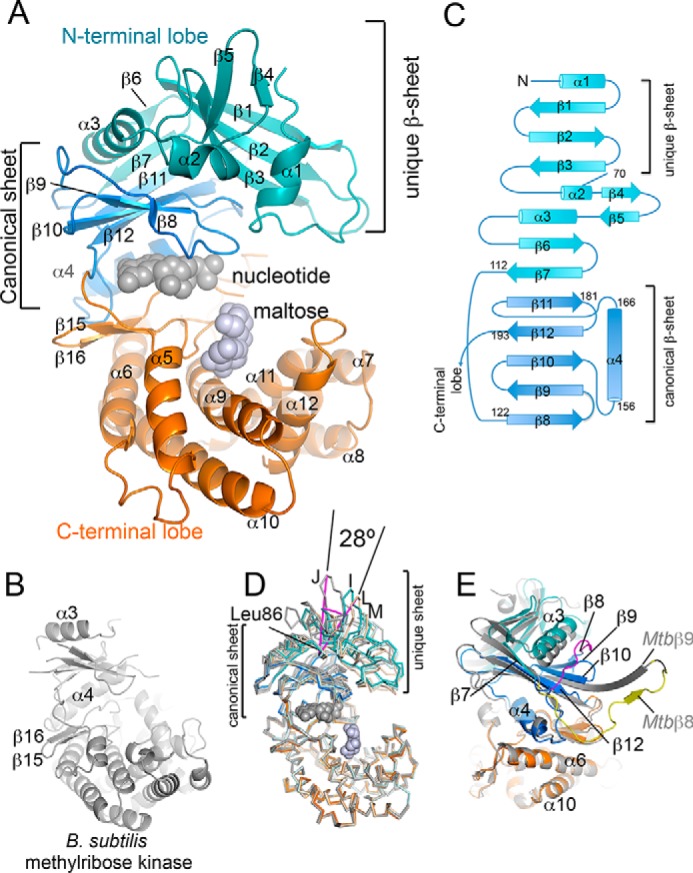
**Structure of *M. smegmatis* Pep2 and topology of its N-terminal lobe.** *A, ribbon diagram* of the *M. smegmatis* Pep2 (*Ms*Pep2) monomer. The conserved and unique regions in the N-terminal lobe are shown in *hues of blue*, matching the color scheme of the topology diagram in *C. Spheres in gray* indicate the binding sites of nucleotide and maltose, as derived from secondary structure-matched superposition with GDP-bound aminoglycoside 2″-phosphotransferase IIIa (PDB code 3TDW (38)) and maltose-bound *M. tuberculosis* Pep2 (PDB code 4O7P (21)), respectively. *B, ribbon diagram* of *B. subtilis* methylribose kinase (PDB code 2PUL (22)), the closest structural neighbor of Pep2 according to DALI (23) (RMSD 3.9 Å for 262 aligned Cα positions, 12% identity). *C,* topology diagram of the N-terminal lobe of *Ms*Pep2. *D,* superposition of *Ms*Pep2 *chains I, J, L*, and *M* with respect to residues 198–400. *E,* superposition of *Ms*Pep2 with *M. tuberculosis* Pep2 (MtPep2) (PDB code 4O7O (21), *gray ribbon*). Secondary structure labels referring to *Mt*Pep2 are preceded by the prefix *Mtb*. The Cα traces deviate following strand β7, where the backbone of *Ms*Pep2 (*magenta*) traverses to the other end of the canonical β-sheet (β8), whereas the backbone of *Mt*Pep2 (*yellow*) extends to the adjacent, NCS-related copy of Pep2 in a domain swap-mediated dimerization.

The Pep2 structure shows the prototypical two-lobe architecture of phosphotransferase enzymes of PFAM family PF01636. Characteristic for this family and that of protein kinase domains (PFAM family PF00069) is the α-helical C-terminal lobe (Fig. 3*A*). This is illustrated by comparison with the closest nonmycobacterial structural neighbor, *Bacillus subtilis* methylribose kinase (PDB code 2PUL (22), RMSD 3.9 Å, 262 aligned Cα, Fig. 3*B*), which superimposes with the C-terminal lobe and parts of the N-terminal lobe of Pep2. However, in Pep2 the N-terminal lobe includes a novel β-sheet at the N terminus (β1–β3), which appears to be unique for mycobacterial Pep2 enzymes, and is missing in structural neighbors identified by distance matrix alignment (DALI (23)), including methylribose kinase. In contrast, the seven-stranded β-sheet preceding the C-terminal lobe (β6–β12) is conserved. We refer to the latter as the canonical β-sheet, although it includes two additional nonconserved strands (β6 and β7) at its N terminus (Fig. 3*C*).

The unique three-stranded β-sheet, which is preceded by a two-turn α-helix, is visible in only a subset of Pep2 protomers (Fig. 3, *A* and *C*), with density lacking between the N terminus and helix α3 (corresponding to residues 1–88) in four of eight Pep2 protomers. Superimposing the Pep2 copies (chains I, J, L, and M), which could be fully traced, with respect to residues 198–400 of the C-terminal lobe results in a close match for the canonical β-sheet of the N-terminal lobe, but it reveals considerable conformational flexibility for the unique β-sheet with a rotation of ∼28° between the two extreme conformers about the “hinge residue” Leu-86 (Fig. 3*D*). Furthermore, we note that the domain swap seen in the structure of *Mtb* Pep2 (21) involves strands of the canonical β-sheet (Fig. 3*E*).

The functional role of the unique β-sheet in Pep2 is not immediately evident from the structure. The nucleotide-binding site resides in the N-terminal lobe, whereas maltose binds opposite to the nucleotide in a pocket of the C-terminal lobe (Fig. 3*A*) (21). Superimposing Pep2 with the ATP-bound structure of *Mvb* Pep2 (PDB code 4WZY (20)) suggests that residues in the unique region of the N-terminal lobe do not directly contact the nucleotide, but one might expect that perturbations of this part of the structure affect nucleotide binding and potentially activity (see “Discussion”).

### Location of active sites

Given that TreS and Pep2 catalyze consecutive reaction steps in the GlgE pathway, it has been suggested that products of the TreS-catalyzed reaction may be channeled directly to the active site of Pep2 (21). However, no such path or channel between the active sites of TreS and Pep2 is apparent in the complex structure. The linear distance from the active site of TreS (chain A) to the nearest maltose-binding site of Pep2 (chain J) measures about 53 Å and traverses the C-terminal lobe of Pep2 (Fig. 2*D* and Fig. S3). The next nearest maltose-binding site (chain I) is 63 Å away, and this time the direct line crosses the hydrophobic core of TreS, again without an obvious opening or tunnel. Within the complex, the active sites of TreS and Pep2 are both open to solvent and do not face each other (Fig. 2*D*), suggesting that following release from TreS, maltose needs to diffuse through solvent on the way to the active site of Pep2.

### TreS:Pep2-binding interface

**Figure 4.**
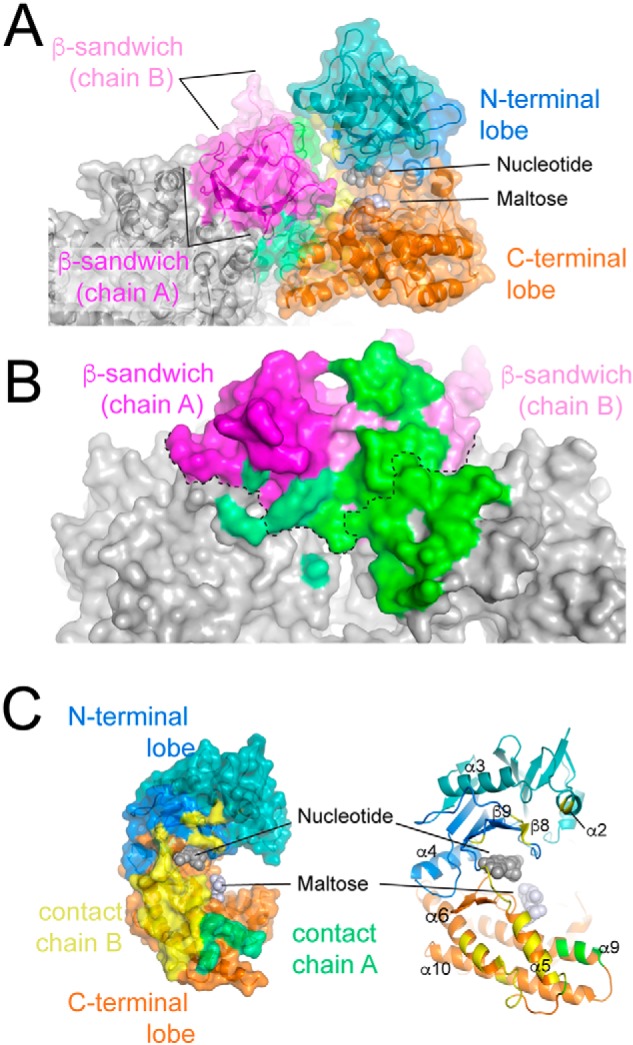
**Contact surfaces between TreS and Pep2.** *A,* side view of the TreS:Pep2 interface for chain I of *Ms*Pep2. The surface of TreS is colored *gray* and in *hues of magent*a (indicating the C-terminal β-sandwich domain of TreS). *Ms*Pep2 is colored in *hues of blue* (N-terminal lobe) and *orange* (C-terminal lobe). Contact surfaces between TreS and Pep2 are colored *green* (on TreS) and *yellow* (on Pep2) according to burial of solvent-exposed surface (calculated using PISA (39)). *B,* area (*green*) on the molecular surface of TreS contacted by chain I of *Ms*Pep2 as seen from the position of the Pep2 subunit. The *dashed line* indicates the boundary between the C-terminal β-sandwich and the TIM-barrel domains of TreS. *C,* area on Pep2 (chain I) contacted by TreS, as seen from the viewpoint of the latter. Areas in *yellow* and *green* contact TreS chains B and A, respectively.

Pep2 forms intimate contacts with the TreS tetramer, revealing a high level of shape complementarity between the binding partners (Fig. 4*A*). Complex formation buries about 1200 Å^2^ of solvent-accessible surface per monomer. The footprint of a single Pep2 subunit covers surface areas belonging to two TreS protomers, including and extending beyond the C-terminal β-sandwich domain(s) (Fig. 4*B*). Both lobes of Pep2 contribute to the contact surface, but the C-terminal lobe provides the lion share (Fig. 4*C* and Fig. S2). Where parts of the N-terminal lobe are disordered, the C-terminal lobe accounts for all observed contacts. Secondary structure elements contributing to the binding interface are helices α5, α6, and α10 in the C-terminal lobe of Pep2, and contacts made by the N-terminal lobe include residues in helix α2, in strand β8, and in the β9–β10 loop (Fig. 4*C*). In addition, contacts also involve the β12–α5 loop, which links the N- and C-terminal lobes. The binding interface is dominated by van der Waals and hydrophobic contacts, corresponding to ∼70% of surface area buried in the interface per Pep2 monomer. In addition, eight hydrogen bonds and two salt bridges (TreS–Arg-312:Pep2–Asp-228 and TreS–Arg-534:Pep2–Asp-218) complement the noncovalent interactions (Table S2).

The binding interface shares structural elements with the nucleotide-binding site, based on the superposition with the ATP-bound structure of *Mvb* Pep2 (PDB code 4WZY (20)) (Fig. S3). According to this superposition, we predict several residues to make contacts with ATP that are part of (or right next to) the binding interface with TreS. Residues in the maltose-binding site (derived from the superposition with maltose-bound *Mtb* Pep2 (PDB code 4O7P (21)) make no direct contacts with TreS.

### Stoichiometry and affinity of the M. smegmatis TreS:Pep2 complex in solution

We had previously investigated the stoichiometry of the complex formed by the *M. tuberculosis* orthologues and found, by analytical ultracentrifugation in equilibrium mode, that the complex encompasses 4 + 4 subunits (*M*_r_ ∼490,000) (14). However, purified *Mtb* Pep2 eluted from a size-exclusion resin as a mix of species (monomers, dimers, and higher oligomers) (14), in contrast to the essentially single-peak elution of monomeric *Msm* Pep2 (Fig. S4). We therefore wished to ascertain whether the self-association behavior affected complexation with TreS.

We initially probed the assembly of the *Msm* TreS:Pep2 complex by size-exclusion chromatography. As was observed previously for the *M. tuberculosis* orthologues, addition of Pep2 to TreS leads to a shift of the TreS tetramer peak to shorter retention times, suggesting complex formation (Fig. S4). We also noticed that the association of *Msm* TreS and Pep2 was pH-dependent. Between pH 7 and 8, the position of the complex peak moved to longer retention times, whereas the absorbance signal corresponding to the Pep2 monomer increased (Fig. S4). In accordance with the stronger association between TreS and Pep2 at mildly acidic pH, the best diffracting crystals were obtained at reservoir pH values between 6.7 and 7.0.

**Figure 5.**
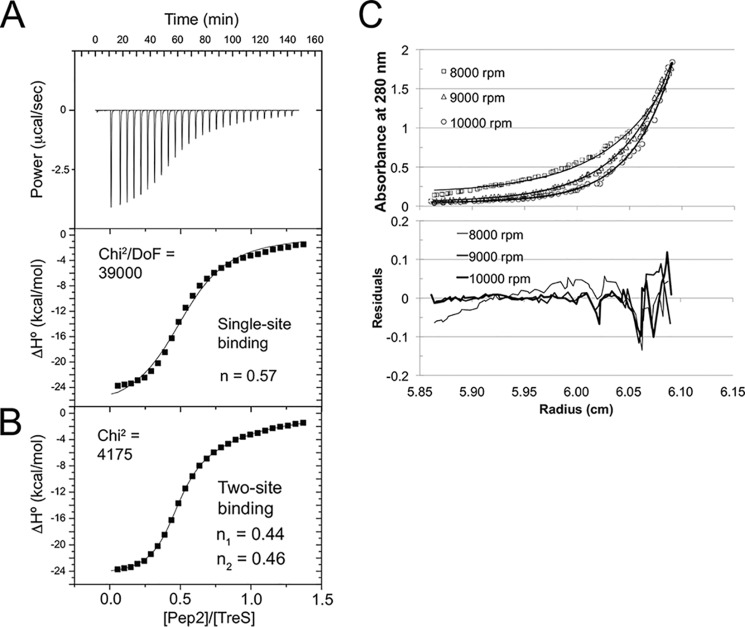
**Probing binding between *M. smegmatis* TreS and Pep2 by isothermal titration calorimetry and sedimentation equilibrium analysis of the *M. smegmatis* TreS:Pep2 complex.** *A* and *B*, proteins were in sodium phosphate buffer at pH values of 6.5, 7.5, and 8.5. The starting concentration of TreS in the reaction chamber was 75 μm, and Pep2 was titrated up to nominal molar ratio of ∼1.5. *A,* trace of the injections at pH 6.5 and plot of integrated peak areas *versus* concentration ratio [Pep2]/[TreS], with the fit representing a single-site binding model. *B,* fit of a two-site binding model to the data in *A. C,* 1:1 molar mixture of *M. smegmatis* TreS and Pep2 was analyzed at three different protein concentrations ([TreS] = 3.75, 2.5, and 1.25 μm) and three rotation speeds (8000, 9000, and 10,000 rpm). Data points are shown as *open symbols*, and *solid lines* represent the best fit (*top panel*) and residuals (*lower panel*), respectively. The fitted mass was 372,823 Da, compared with a calculated mass of 370,407 Da for a complex of 4 TreS + 2 Pep2. Data shown illustrate the fit for the highest protein concentration (see also Fig. S5).

To determine the affinity between *Msm* TreS and Pep2, we measured binding by isothermal titration calorimetry (ITC), buffering with phosphate, and titrating Pep2 to TreS up to a molar ratio [Pep2]/[TreS] of about 1.5. Setting the initial concentration of TreS at 75 μm, we measured binding isotherms at 25 °C at pH 6.5, 7.5, and 8.5 (Table 1 and Fig. 5*A*). Fitting a single-site binding model, the strongest affinity was measured at pH 6.5 with a *K_d_* of 3.5 μm, increasing to 34.8 μm at pH 8.5 (Table 1). In terms of standard Gibbs energy, Δ*G*^0^, the change is about 16%. Importantly, the ITC data also allow one to derive the stoichiometry of the interaction, *n*, which was about 0.5 for pH 6.5 and 7.5 (Table 1). As Pep2 was titrated to TreS, which forms a stable tetramer in solution (14), a value of *n* = 0.5 means that the TreS tetramer binds two copies of Pep2. As the affinity decreased with rising pH, *n* dropped to 0.22, suggesting a single copy of Pep2 is binding to the TreS tetramer at pH 8.5. Finally, we tested whether the binding affinity was sensitive to the ionic strength of the buffer, and we found that the *K_d_* value remained unchanged between 200 and 400 mm NaCl (Table S3).

**Table 1:** **ITC data of the TreS:Pep2 interaction** The sample chamber contained 75 μm *M. smegmatis* TreS at the start of the titration, and Pep2 or Pep2-Δ70 was titrated to TreS up to a molar ratio of about 1.5 of [Pep2]/[TreS].

	Pep2 (wildtype)			Pep2-Δ70
pH	6.5	7.5	8.5	6.5
*T* (K)	298	298	298	298

**Single-site binding model**				
*K_a_* (10^5^ m^−1^)	2.87 ± 0.26	1.60 ± 0.12	0.287 ± 0.03	0.35 ± 0.08
*K_d_* (μm)	3.48	6.25	34.8	26.8
Δ*G*^0^ (kJ/mol)	−31.1	−29.7	−25.4	−25.9
Δ*H*^0^ (J/mol)	−131,000	−78,400	−93,200	−74,900
*T*Δ*S*^0^ (J/mol)	−99,400	−48,700	−67,600	−49,000
*n*	0.574 ± 0.007	0.459 ± 0.008	0.22 ± 0.04	0.681 ± 0.004

**Two-site binding model**				
*K_a_*_, 1_ (10^5^ m^−1^)	19.7 ± 3.2			
*K_a_*_, 2_ (10^5^ m^−1^)	0.55 ± 0.08			
*K_d_*_, 1_ and *K_d_*_, 2_ (μm)	0.5, 18			
*n*_1_ and *n*_2_	0.445 ± 0.004, 0.457 ± 0.055			

We corroborated the apparent 2:1 stoichiometry of the *Msm* TreS:Pep2 complex by analytical ultracentrifugation in sedimentation equilibrium mode, comparing the sedimentation of the *Msm* TreS tetramer to an equimolar molar mixture of *Msm* TreS and Pep2. Fitting a single-species model against data from three protein concentrations (6, 4, and 2 μm) and three different rotor speeds (Fig. 5*C* and Fig. S5), the mass determined for *Msm* TreS was 248,550 Da, compared with the calculated mass of the *Msm* TreS tetramer of 272,000 Da. A 1:1 molar mixture of TreS and Pep2 (3.75, 2.5, and 1.25 μm with respect to monomers) analyzed in the same way resulted in a fitted mass of 372,823 Da. Given calculated masses of 272,000 Da for the TreS tetramer and 48,800 Da for the *Msm* Pep2 monomer, and considering that *Msm* Pep2 elutes predominantly as a monomeric species from the size-exclusion resin (Fig. S4), the data strongly suggest that the sedimenting complex is the TreS tetramer binding two copies of Pep2, which is a “4 + 2” complex.

### Enzymatic activity of Msm Pep2

**Figure 6.**
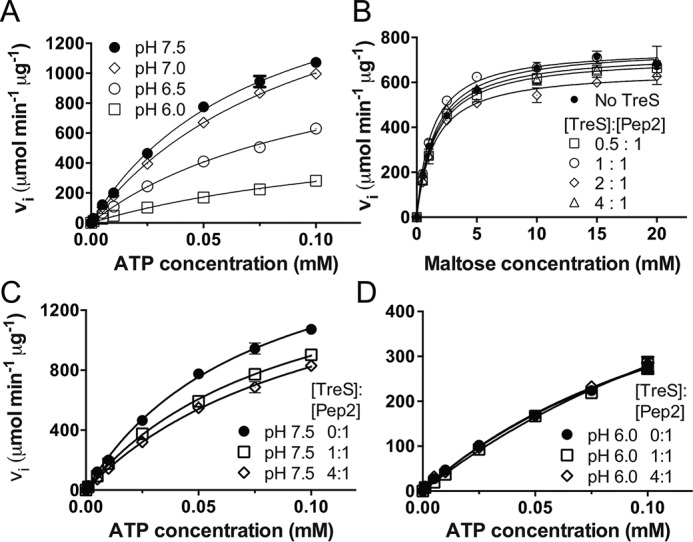
**Activity of *M. smegmatis* Pep2 as a function of pH and in presence/absence of *M. smegmatis* TreS.** Activity of Pep2 was analyzed, coupling generation of ADP to depletion of NADH (see under “Experimental procedures”). *A,* pH dependence of activity in the presence of 20 mm maltose. *B,* Pep2 activity at pH 6.0 as a function of maltose concentration (0.3 mm ATP) and adding TreS at molar ratios as indicated. *C* and *D*, probing the effect of *M. smegmatis* TreS on Pep2 activity at pH 7.5 and 6.0, respectively, with maltose at 20 mm. Data in *A–D* were fitted to the Michaelis-Menten equation (*v_i_* = *V*_max_ [S]/(*K_M_* + [S])). *Error bars* are omitted where they appear smaller than the corresponding data point marker.

We had observed previously that activity of *Mtb* Pep2 increased markedly in the presence of *Mtb* TreS (14). We tested whether this was also the case for *Msm* Pep2. Given the pH-dependent affinity between *Msm* Pep2 and TreS, we first tested how activity responds to pH, and we found that it declined significantly as pH decreased (Fig. 6*A* and Table 2). Yet even at the lowest pH, *Msm* Pep2 activity was still several orders of magnitude higher than that of *Mtb* Pep2 in terms of *V*_max_ at the same assay conditions (*V*_max_ corrected for enzyme concentration, *cf*. Table 2 in Roy *et al.* (14)). At pH 6.0, *Msm* Pep2 activity was largely indifferent to adding *Msm* TreS when varying maltose (ATP at 0.3 mm, Fig. 6*B*). In the converse experiment, adding *Msm* TreS at molar ratios of 1:1 or 4:1 (TreS:Pep2) to *Msm* Pep2 slightly depressed activity at pH 7.5 but had virtually no effect at pH 6.0 (Fig. 6, *C* and *D*). Thus, the rate enhancement effect observed for the *Msm* TreS:Pep2 complex does not appear to hold for the *M. smegmatis* orthologues, although the latter is more active than the former by several orders of magnitude.

**Table 2:**
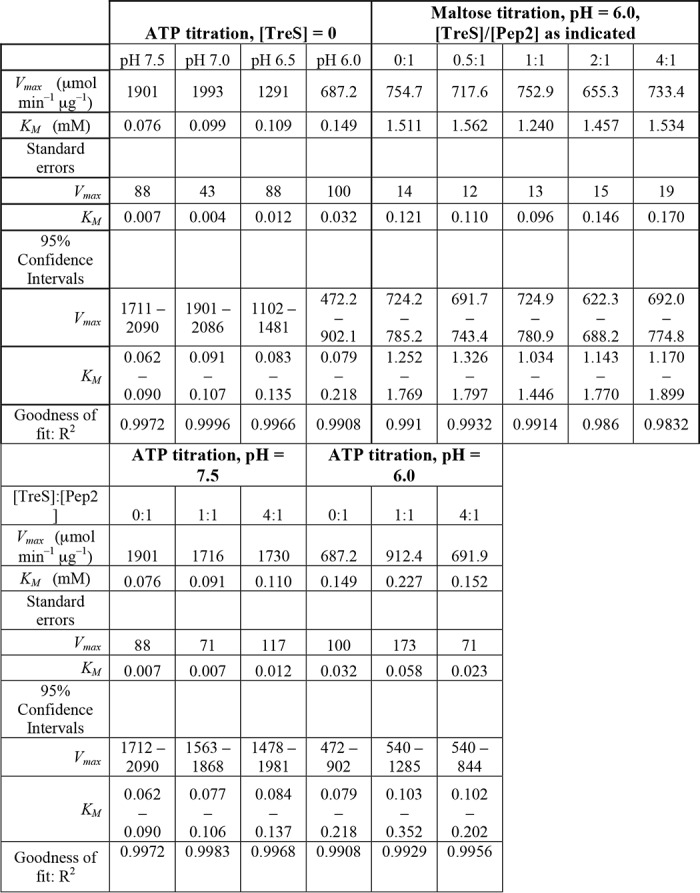
**Enzymatic activity of *M. smegmatis* Pep2** Activity was probed through a coupled assay, spectroscopically monitoring NADH depletion (see “Experimental Procedures”). Maltose was at 20 mm when titrating ATP, and ATP was at 0.3 mm when titrating maltose.

### Is the M. smegmatis complex structure representative of Mtb TreS:Pep2?

**Figure 7.**
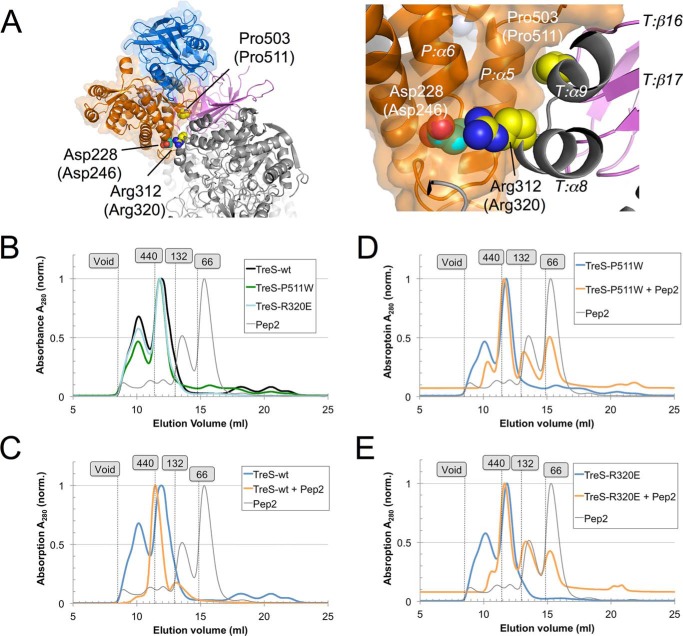
**Probing *M. tuberculosis* TreS:Pep2 complex formation with amino acid substitutions at the binding interface.** *A,* location of the substitution sites *Msm* Pro-303 (*Mtb* Pro-311) and *Msm* Arg-312 (*Mtb* Arg-320), with a close-up view on the *right*. Pep2 is shown with a translucent molecular surface (*blue* and *orange ribbon*), and TreS is shown as a *ribbon* only (*gray* and *purple*). Residue numbers refer to the *Msm* sequences, with corresponding *Mtb* residue numbers in *parentheses*. Selected secondary structure elements are labeled, with letters *T* and *P* indicating TreS and Pep2, respectively. *B,* size-exclusion profiles of *Mtb* TreS (WT, P511W, and R320E) and of Mtb Pep2 in the absence of their complex partners. Void volume and position of markers ferritin (11.4 ml) and albumin (14.8 ml, 13 ml) are indicated. *C–E*, size-exclusion profiles of *Mtb* TreS (WT, P511W, and R320E) in the presence of an equimolar amount of *Mtb* Pep2 superimposed over the elution traces of free TreS of *B*. For the ease of comparing separate runs, the absorbance signals were scaled such that the maximal absorbance is indicated as 1.0. To facilitate a direct comparison between the elution behavior of TreS:Pep2 complex (*orange*) to that of the constituent proteins, the elution traces of Pep2 (*gray*) and of the relevant forms of TreS (WT or mutant) of *B* appear again in *C–E* (*blue*).

The discrepancy between the TreS:Pep2 complexes of *M. smegmatis* and *M. tuberculosis* in terms of solution stoichiometry and enzymatic behavior upon complex formation prompted the question whether the present crystal structure is representative of the TreS:Pep2 complex of *M. tuberculosis*. Examining the binding interface, we found that the two salt bridges (TreS–Arg-312:Pep2–Asp-228 and TreS–Arg-534:Pep2–Asp-218) link residues that are conserved on either side of the interface. In addition, a conserved proline residue in TreS (Pro-503), located in the loop between strands β16 and β17, is packing tightly against backbone atoms of helix α5 of Pep2 (Fig. 7*A*). We reasoned that introducing a bulky side chain at the Pro-503 site or swapping the basic side chains (Arg-312 and Arg-534) for acidic amino acids should be sufficient to destabilize complex formation, given the moderate overall affinity (*K_d_* = 3.5 μm). Although *Mtb* TreS–R536E (*Msm* Arg-534) did not express well enough, sufficient amounts of protein could be made of *Mtb* TreS–P511W (*Msm* Pro-503) and TreS–R320E (*Msm* Arg-312). The mutant proteins showed the same elution behavior on a Superdex 200 size-exclusion resin as WT *Mtb* TreS (Fig. 7*B*, peaks at 10.2 and 11.7 ml), whereby the peak at 11.7 ml likely represents the TreS tetramer (280 kDa) as it is located between the ferritin marker (440 kDa) and the dimer peak for albumin (132 kDa). Thus, this comparison indicates that the mutant TreS proteins maintain the tertiary structure of the WT enzyme.

To probe Pep2 binding, we incubated *Mtb* TreS (WT and mutants) with an equimolar amount of *Mtb* Pep2 and analyzed the protein mixture by size-exclusion chromatography (Fig. 7, *C*, *D*, and *E*). Only when incubated with WT *Mtb* TreS did *Mtb* Pep2 drive a distinct shift of the main TreS elution peak at 11.8 ml (Fig. 7*C*) to 11.4 ml, coinciding with the 440-kDa ferritin marker. This peak contains both proteins (Fig. S6). In contrast, incubation with either mutant (P511W and R320E, Fig. 7, *D* and *E*) resulted only in a minor shift of the TreS tetramer peak (11.7 ml). The peak representing the Pep2 monomer (15.5 ml) is eliminated in the incubation with WT TreS (Fig. 7*C*), but it is rather prominent when Pep2 is incubated with either mutant (Fig. 7, *D* and *E*). This differential behavior clearly indicates that Pep2 binding to the mutant TreS proteins is perturbed by introducing mutations at the TreS:Pep2 interface, thus providing evidence that the *Msm* TreS:Pep2 complex structure is representative of its counterpart in *M. tuberculosis.*

## Discussion

Noncovalent complexes between enzymes that catalyze successive reaction steps in metabolic pathways provide a means for cells to regulate synthesis of metabolites and to efficiently exploit limited nutritional resources (24). This aspect is especially relevant for *M. tuberculosis*, an organism that, for the most part, resides sequestered in host macrophages, where it faces a generally nutrient-poor and in particular a carbohydrate-poor environment (25). Although the α-glucan capsule is shed readily in culture, previous evidence has implicated α-glucan synthesis in the ability *of M. tuberculosis* to persist in the host (4, 6), and thus regulatory features of pathways linked to capsule synthesis are likely pertinent for the environmental niche occupied by *M. tuberculosis*.

Complex formation between TreS and Pep2 is mirrored by TreS:Pep2 fusion enzymes in a sizeable subset of microbial species (96 bacterial and 6 archaeal as listed in Ref. 9). The TreS:Pep2 complex structure is compatible with a fused protein in that the stoichiometry in the crystallized complex is 1:1 and the C-terminal β-sandwich domain of TreS is the docking site for Pep2. The linear distance between the C terminus of TreS and the nearest ordered N-terminal residue of Pep2 is ∼40 Å, a distance compatible with sequence insertion in TreS:Pep2 fusion from *Pseudomonas aeruginosa*, which comprises an additional 16 or 30 residues between the termini of TreS and Pep2 relative to the *M. tuberculosis* and *M. smegmatis* orthologues, respectively (Fig. S7).

The architecture and biochemical behavior of the TreS:Pep2 complex has parallels to the complex of the mycobacterial chorismate mutase (CM) bound to 3-deoxy-d-arabino-heptulosonate-7-phosphate synthase (DAHP synthase) of the shikimate pathway of aromatic amino acid biosynthesis (PDB code 2W19 (26)). As is the case for TreS, the catalytic domain of DAHP synthase folds as an α/β- or TIM-barrel and exists as a tetramer in solution. In the hetero-octameric DAHP synthase:CM complex, pairs of CM attach to opposite corners of the DAHP synthase tetramer (Fig. S8). Importantly, association of CM with DAHP synthase results in strongly-amplified mutase activity (26, 27). Rate amplification of mutase activity was attributed to a re-arrangement of active-site residues upon CM binding to DAHP synthase, leading to a molecular environment favoring catalysis (26).

For TreS:Pep2, the level of rate enhancement is modest in *M. tuberculosis* (*cf*. Table 2 in Ref. 14) and is essentially absent in *M. smegmatis*, at least under *in vitro* conditions (Fig. 6, *C* and *D*, and Table 2). Given that the individual proteins align with high levels of sequence identity (83% TreS and 66% Pep2), and given the diminished or abrogated binding of Pep2 when the *Mtb* TreS:Pep2 interface is perturbed (Fig. 7), it is unlikely that complexes from *M. tuberculosis* and *M. smegmatis* differ in architecture, even though the domain-swapped dimeric structure of *M. tuberculosis* Pep2 (21), possibly a crystallization artifact, confounds the comparison. The domain-swapped *Mtb* Pep2 dimer is incompatible with the 4 + 4 architecture, as significant steric overlap occurs when superimposing a protomer of the *Mtb* Pep2 dimer onto, for instance, chain I of Pep2 in the *Msm* TreS:Pep2 complex (Fig. S9). Domain swapping is not seen in the stand-alone structure of *Mvb* Pep2 (20) (61% identity to *Mtb* Pep2) nor is it occurring in *Msm* Pep2 in the context of the *Msm* TreS:Pep2 complex. Also, the size-exclusion data clearly show a mixed population of *Mtb* Pep2 in solution that clearly includes a monomeric species (Fig. 7*B*). Finally, we lack structures of stand-alone *Msm* Pep2 and of the *Mtb* TreS:Pep2 complex.

Notwithstanding the caveats outlined above, the present complex structure gives hints as to how complex formation could affect reaction rates. First, four of the eight Pep2 molecules present in the asymmetric unit display a partially disordered N-terminal lobe, which accommodates the ATP-binding site. This observation suggests inherent structural flexibility of this region, possibly affecting activity. Indeed, a 70-amino acid N-terminal truncation (*Msm* Pep2-Δ70, Fig. S10) demonstrates substantially reduced maltokinase activity and affinity to TreS. In size-exclusion chromatography, Pep2-Δ70 showed a diminished shift of the complex peak to shorter retention times, whereas the dissociation constant, *K_d_*, increased from 3.5 to 28.6 μm (Table 1). Notably, the Δ70-truncation reduced *V*_max_ 10-fold, from 2513 (± 205) to 242 (± 41) μmol min^−1^ mg^−1^ (Fig. S10).

Second, the complex structure demonstrates that the ATP-binding site shares structural elements with the TreS:Pep2 interface (Fig. 4*C* and Fig. S3). Identifying residues involved in ATP binding by mapping the structure of ATP-bound *Mvb* Pep2 (PDB code 4WZY (20)) onto Pep2 (chain I) of the TreS:Pep2 complex demonstrates a high level of sequence conservation for residues within a 4 Å distance cutoff of ATP (Fig. S2), and it identifies secondary structure elements that are part of the TreS:Pep2 interface and, at the same time, contributes residues to the ATP-binding site. These include the β8–β9 loop, the β12–α5 loop, which links N- and C-terminal lobe, and strand β15 (Fig. S3). In contrast, the maltose-binding site, identified by alignment with maltose-bound *Mtb* Pep2 (PDB code 4O7P (21)) is completely conserved for contact residues within a 4 Å distance cutoff (Figs. S2 and S3). Furthermore, the maltose-binding site includes far fewer structural elements sharing in the TreS:Pep2 interface and contributing contacts to maltose. Thus, we may attribute the modest rate enhancement seen in the *M. tuberculosis* TreS:Pep2 complex to stabilization of the N-terminal lobe of Pep2 when bound to TreS.

We previously provided evidence that complex formation between TreS and Pep2 occurs *in vivo* (14). However, the moderate affinity for binding of Pep2 to TreS (*K_d_* = 3.5 μm) suggests that the TreS:Pep2 complex is only transient rather than constitutive (24). This notion is also consistent with the predominant stoichiometry of the *Msm* TreS:Pep2 complex in the solution state (TreS_4_:Pep2_2_). Furthermore, it suggests that even as a fusion protein, noncovalent association between the TreS and Pep2 domains may not be constitutive. Such behavior would resemble endoglucanase EngD from *Clostridium cellulovorans,* where the crystal structure shows noncovalent, intramolecular association between the C-terminal carbohydrate-binding domains and the catalytic domains, but small-angle x-ray scattering analysis demonstrated that the carbohydrate-binding domains do not constitutively associate with the catalytic domains in the solution state (28).

The discrepancy in complex stoichiometry between the crystallized complex and the behavior in solution is likely explained by crystal lattice contacts, which can influence the thermodynamic equilibrium by (partial) exclusion of solvent and introduction of packing interactions that do not exist in the solution state. Close inspection of the ITC data showed systematic albeit subtle deviations between the single-site binding model and the data points (Fig. 5*A*). Introducing a two-site binding model removes the systematic deviation (Fig. 5*B*) and suggests that the two sites bind with *K_d_* values of 0.5 μm (site 1) and 18 μm (site 2), respectively (Table 1). The stoichiometry for site 1 remains near 0.5 (*n*_1_ = 0.45), whereas for site 2, the stoichiometry is *n*_2_ = 0.46. Thus, overall the stoichiometric ratios of the two sites add up to 0.91, *i.e.* near parity between the complex components, and the difference in magnitude between *K_d_*_, 1_ and *K_d_*_, 2_ could account for the apparent preponderance of a 4 + 2 configuration in solution.

**Figure 8.**
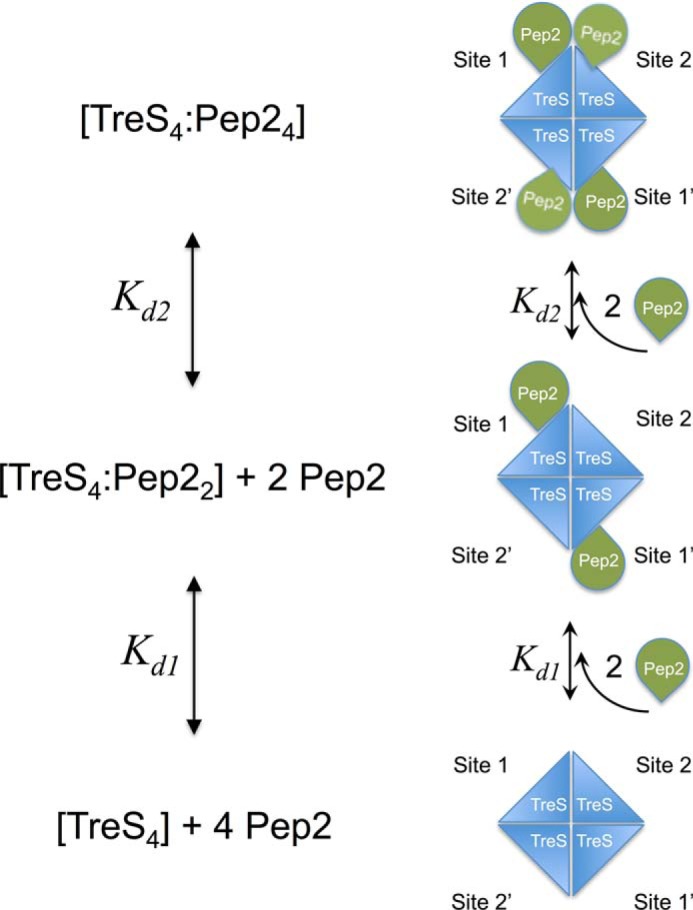
**Illustration of the two-site binding model for binding of four copies of Pep2 to the TreS tetramer.** Binding of the first copy of Pep2 at an unoccupied apical binding site (*Site 1*) is governed by *K_d_*_,__1_ and does not influence affinity for binding of the first copy at the opposite apex (*Site 1′*). Hence, Sites 1 and 1′ have identical dissociation constants. Binding of the second copy of Pep2 may encounter steric constraints imposed by the previously bound copy, and therefore, the affinity for binding the second copy, *K_d_*_,__2_, at *Site 2* (or *Site 2′)* is different.

Adding parameters to a mathematical model is bound to result in a more accurate description of the observed data. However, the crystal structure elegantly rationalizes the binding behavior observed in the ITC experiment. We propose that the two distinct binding sites (with different affinities) correspond to binding the first and second copy, respectively, of Pep2 to the same apex of the TreS tetramer (Fig. 8). The binding sites for pairs of Pep2 monomers at opposite apices of the TreS tetramer are spatially well separated (Fig. 2, *A* and *B*). Thus, binding of the first copy at one apex (site 1) does not geometrically constrain binding of the first Pep2 copy at the opposite apex (site 1′, Fig. 8), and thus binding to sites 1 and 1′ occurs with the same affinity. However, once an apical position is occupied by a single copy of Pep2, binding of the second copy may be hindered by the first copy, and this may lead to an increase of *K_d_* values for the second binding event. Indeed, the conformations of the bound Pep2 copies are not identical. By :Superimposing Pep2 chains I and J, which are fully ordered and occupy the same apex of the TreS tetramer (Fig. 2*B*), it emerges that their N-terminal lobes do not match well (Fig. 3*D*). Forcing the two adjacent copies into an identical conformation results in steric hindrance between their N-terminal lobes. When the N-terminal lobes are truncated (as in Pep2-Δ70), the stoichiometry determined in the ITC experiment converges to *n* = 0.68, (Table 1), suggesting that the 4 + 2 configuration is no longer dominant.

In conclusion, we have provided a detailed structural model of the complex formed between trehalose synthase and maltokinase of mycobacteria, with mutagenesis data indicating that this complex structure is a valid representative of the *Mtb* TreS:Pep2 complex. The structure rationalizes rate enhancement of Pep2 induced by binding of Pep2 to the TreS tetramer and together with the example of the DAHP synthase:CM complex points to complex formation as a mechanism that can contribute to regulating pathway activity in mycobacteria.

## Experimental procedures

### Cloning of M. smegmatis Pep2, TreS

The DNA sequences of TreS (MSMEG_6515) and Pep2 (MSMEG_6514) were cloned from *M. smegmatis* genomic DNA by polymerase chain reaction (PCR) using the following primers: TreS forward, GATCGATC**CATATG**GAGGAGCACACGCAGGGCAG, and TreS reverse, GATCGATC**AAGCTT**CATTGCTGGCGCTCCCGGTTCTGGATC; and Pep2 forward, GATCGATC**CATATG**AGTGTCGAATTCGAGGACTGGC, and Pep2 reverse, GATCGATC**AAGCTT**CACTCCAGAAGACGTGCGATGGAG. The PCR products were ligated into the NdeI and HindIII sites (underlined) of plasmid vector pET28a (Novagen), and the expressed proteins included an N-terminal His_6_-affinity tag.

### Cloning of M. smegmatis Pep2-Δ70

N-terminally truncated *Msm* Pep2 was cloned from a pET28a vector containing MSMEG_6514 (*M. smegmatis pep2*) using the Phusion High-Fidelity DNA polymerase kit (New England Biolabs, UK), introducing a STOP codon at the C terminus. The primers were as follows: Δ70 forward, GATC**CATATG**GTCGCCACGATCGGCATCGCCGAC, and Δ70 reverse, GATC**AAGCTT**TCACTCCAGAAGACGTGCGATGGAGCCCAG. The purified PCR product (QIAquick extraction) was ligated into the NdeI and HindIII restriction sites of plasmid vector pET28a, yielding a construct encoding N-terminally His_6_-tagged Pep2-Δ70.

### Protein production

*Escherichia coli* BL21 (DE3) cells were heat-transformed with plasmids, encoding either *Msm* TreS or Pep2, and cultured onto agar plates (LB/kanamycin 25 μg/ml). A single colony was used to inoculate 10 ml of LB broth and 1% (w/v) glucose and kanamycin (25 μg/ml), incubated overnight (37 °C), and propagated to bulk cultures of Terrific Broth media (29) (kanamycin at 50 μg/ml, 37 °C, 180 rpm). At *A*_600_ = 0.4–0.6, the cultures were cooled to 16 °C (3 h), and protein expression was induced using 0.6 mm IPTG (TreS) or 0.1 mm IPTG (Pep2) and incubated (12–16 h, 16 °C, 180 rpm). For Pep2 expression, 10 mm maltose (final concentration) was added to the culture medium prior to induction with IPTG. Cells were harvested (7000 × *g*, 10 min, 4 °C), washed with phosphate-buffered saline (PBS), and resuspended in lysis buffer (30 ml, 25 mm HEPES-NaOH, pH 7.6, 10% (v/v) glycerol), including either 0.3 m (TreS) or 1 m (Pep2) NaCl. Pellets were frozen (−80 °C), thawed, and supplemented with protease inhibitor mixture (Roche Applied Science), 1 mm phenylmethylsulfonyl fluoride, 10 mm MgCl_2_, and 10 μg/ml DNase I pellet, as well as maltose (for Pep2 lysate, 100 mm final concentration), followed by passage (four times) through a French press (Thermo Spectronic FA-078). The cleared lysate (27,000 × *g*, 30 min, 4 °C) was diluted 4-fold with buffer A (25 mm HEPES-NaOH, pH 7.6, 10% (v/v) glycerol), filtered (0.45-μm pore size), and loaded on a pre-equilibrated Ni-NTA column (5 ml, GE Healthcare).

The column matrix was washed with buffer A and with 20, 40, and 60 mm imidazole in buffer A, respectively. The protein was eluted with 500 mm imidazole in buffer A, and fractions were analyzed by SDS-PAGE. The eluate was diluted 20-fold with buffer B (20 mm BisTris, pH 6.0, 10% (v/v) glycerol for TreS, 50 mm MES, pH 5.8, 10% (v/v) glycerol for Pep2), filtered (0.45 μm), and applied on a HiTrap Q-column (1 ml, GE Healthcare) pre-equilibrated with 20 mm BisTris, pH 6.0, 50 mm NaCl (for TreS) or 50 mm MES, pH 5.8, 100 mm NaCl (for Pep2). The column was washed with buffer B supplemented with NaCl (50–500 mm, increments of 50 mm). Fractions were analyzed by SDS-PAGE and pooled, followed by concentration in Amicon Ultra-4 centrifugal filter units, and then loaded onto a HiPrep Sephacryl 26/60 S-300HR column (GE Healthcare). Fractions containing protein were concentrated as described before. The purification of Pep2-Δ70 followed the protocol of WT Pep2, except that NaCl was set to 300 mm prior to ion exchange chromatography, rather than 50 mm.

### Crystallization and structural determination

Crystals of the TreS:Pep2 complex were grown by vapor diffusion in 96-well plates, using a Mosquito liquid handling system (TTP Labtech) to set up crystallization drops (100 nl of protein + 100 nl of reservoir solution) containing mixtures of *M. smegmatis* TreS and Pep2, at molar ratios between 1:1 and 1:2. Well-formed crystals grew over a reservoir of 9–10% PEG 8000, 4% v/v glycerol, 200 mm MgCl_2_, and 0.1 m Tris-HCl, pH 6.7–7.0. Crystals were immersed briefly in mother liquor supplemented with 15–20% (v/v) glycerol, and flash-frozen in liquid nitrogen. Diffraction data (Table S1) were processed using XDS/XSCALE (30). Phases were calculated by molecular replacement (MR) (PHASER (31)) using PDB code 3ZO9 (19) as the initial search model.

The first of two copies of the TreS tetramer could be placed using a tetrameric search model (PDB code 3ZO9, PHASER, *Z* score >30 after translation search), whereas the second TreS tetramer was placed by searching with a single TreS subunit and then deriving positions of the other three monomers by applying NCS operators. The individual protomers were adjusted into clearly visible secondary structure density by real-space rigid-body fitting (COOT (32)).

An initial model for Pep2 was generated using the HHpred threading server (https://toolkit.tuebingen.mpg.de/#/tools/hhpred^6^ (33)) and the MODELLER software (34), covering residues 100–440 of the Pep2 sequence. A first copy of Pep2 was placed by MR (PHASER, *Z* score ∼11), which matched secondary structure density features that had appeared by phasing with TreS alone. Further copies of Pep2 were placed in a cyclic fashion, applying NCS operators, then real space rigid body fitting, refining the combined model in REFMAC5, and reiterating the cycle. After placing three copies of Pep2, the resulting σ_A_-weighted 2*F_o_* − *F_c_* density map displayed additional secondary structure elements not covered by the Pep2 model, revealing the location of helix α3 and strands β1–β3, respectively. Iterative rounds of chain tracing, model rebuilding and refinement (COOT (32), REFMAC5 (35), and PHENIX.REFINE (36)) allowed us to construct a model comprising two TreS tetramers and the backbone of eight Pep2 monomers. In the later stages of building this model, we obtained access to the coordinates of Pep2 from *M. tuberculosis* (PDB code 4O7O (21)) and *M. vanbaalenii* (PDB code 4U94 (20)), which helped to correct the sequence register and to dock side chains onto the backbone structure where justified by density. We applied tight NCS restraints throughout and modeled atomic displacements by refining TLS parameters (one set per protomer) and grouped B factors. Refinement statistics are shown in Table S1. Structure factors and coordinates for the crystal structure of *M. tuberculosis* TreS:Pep2 are deposited in the PDB under accession code 5JY7.

### Size-exclusion chromatography

The elution of equimolar molar mixtures of TreS:Pep2 from a Sephacryl S-300HR resin (320-ml column volume) was assayed at pH 6.0–9.0, monitoring UV absorbance at 280 nm with a flow rate of 0.5 ml/min, and loading the column with an initial concentration of 30 μm for each protein. The proteins were buffered in 50 mm sodium phosphate, 300 mm NaCl, 10% (v/v) glycerol. The pH value was set by choosing appropriate ratios of mono- to dibasic sodium phosphate. Fractions (5 ml) were analyzed by SDS-PAGE. For the elution of Pep2-Δ70 from the same size-exclusion matrix, the protein was in 50 mm MES buffer, pH 6.0, 500 mm NaCl, and 10% (v/v) glycerol. The association of Pep2-Δ70 with TreS was analyzed in 50 mm sodium phosphate, pH 6.5, 300 mm NaCl, 10% (v/v) glycerol.

### Analytical ultracentrifugation

Sedimentation equilibrium was carried out in a Beckman Optima XL-A analytical ultracentrifuge equipped with absorbance optics. Protein samples were dialyzed overnight in 50 mm sodium phosphate, pH 6.5, 300 mm NaCl, and 10% (v/v) glycerol. Equilibrium experiment was performed using six-channel Epon centerpieces with quartz windows at a rotor temperature of 4 °C for a total duration of 116 h. Absorbance data were recorded at 280 nm at rotation speeds of 8000, 9000, and 10,000. At each rotation speed, the sample was allowed to reach equilibrium during a 24-h period. Finally, the sample was run at 25,000 rpm for 20 h. The data were analyzed using SEDPHAT (37). Parameters for solvent density and viscosity and for the partial specific volume of the proteins were calculated using SEDNTERP.

### Activity assay

To monitor phosphorylation of maltose, conversion of ATP to ADP was enzymatically coupled to oxidation of NADH (via pyruvate kinase and lactate dehydrogenase), and the latter was monitored fluorometrically (excitation 340 nm and emission 450 nm). Fluorescence units were converted to concentrations of ADP by an internal calibration curve for NADH. The assays were performed in triplicate in 96-well plates, using a BMG PHERAstar FS microtiter plate reader, and MARS and GraphPad Prism software to record and analyze data, respectively.

Reaction mixtures contained 50 mm sodium phosphate, 300 mm NaCl, 10% (v/v) glycerol, 10 mm MgCl_2_, adjusting the pH by mixing mono- and di-basic buffer in appropriate ratios. Enzyme concentrations were 0.45-1.0 μm for WT Pep2 or 1-.35 μm for the mutant Pep2-Δ70. When varying ATP, maltose was present at 20 mm, and ATP was at 0.3 mm when varying maltose. The coupling reagents were present at these initial concentrations: 4 mm phosphoenolpyruvate, 2 units of pyruvate kinase, 2 units of lactate dehydrogenase, and 0.1 mm NADH.

ITC experiments were performed using a VP-ITC microcalorimeter from MicroCal, LLC (Northampton, MA). Both proteins, TreS and Pep2, were dialyzed against 50 mm sodium phosphate, pH 6.5–8.5 (mixing mono- and dibasic phosphate), 300 mm NaCl, and 10% (v/v) glycerol. Prior to the experiment, the buffered protein solutions were degassed while incubating at 2 °C below the experimental temperature (25 °C). A total volume of 288 μl of Pep2 (at 500 μm in the syringe) was titrated into 1.4 ml of TreS (75 μm initial concentration with respect to monomer). A total of 29 injections (10 μl each time) were made, with a 300-s interval between injections. The data were analyzed using the Origin 7.0 software package (Microcal, Northampton, MA). The experimental data were fitted to models describing either one or two binding sites.

Site-directed mutants of *Mtb* TreS were generated by *de novo* gene synthesis (Genescript), and the resulting DNA sequences (codon-optimized for expression in *E. coli*) were subcloned into pET28a plasmids containing a kanamycin resistance cassette. Plasmids were verified by sequencing and heat -hock transformed into *E. coli* BL21(DE3) cells, and transformants were cultured on LB agar plates selecting for kanamycin resistance (50 μg/ml kanamycin, 37 °C, overnight). Proteins were purified from cell extracts as described under “Protein production,” but replacing the ion-exchange with a size-exclusion column (Superdex 200 Increase 10/300 GL, GE Healthcare). Fractions of the Ni-NTA eluates containing the His-tagged proteins were pooled, concentrated to a volume of ≤1 ml (Amicon Ultra, >10 kDa), and applied to the Superdex column, which was pre-equilibrated in 50 mm NaPO_4_, pH 6.5, 100 mm NaCl, and 10% v/v glycerol, with buffer exchange occurring through the size-exclusion step.

Complex formation between *Mtb* Pep2 and *Mtb* TreS (WT, or mutants P511W and R320E) was probed by size-exclusion chromatography (Superdex 200 Increase 10/300 GL, bed volume 24 ml) and monitoring UV absorbance at 280 nm. Prior to loading the column, equimolar amounts of *Mtb* Pep2 and *Mtb* TreS (WT or mutants) were mixed, incubated on ice for 30 min, then loaded onto the column, and eluted with a flow rate of 0.5 ml/min for a total elution volume of 30 ml. Elutions from the column were calibrated using bovine albumin (66 kDa) and ferritin (440 kDa).

## Author contributions

A. A. K., G. S. B., and K. F. conceptualization; A. A. K., T. R. P., and K. F. formal analysis; A. A. K., R. R., C. G., K. I. K., T. R. P., L. J. A., and K. F. investigation; A. A. K., T. R. P., L. J. A., G. S. B., and K. F. writing-review and editing; R. R. and L. J. A. resources; G. S. B. and K. F. funding acquisition; K. F. data curation; K. F. writing-original draft; K. F. project administration.

## Supplementary Material

Supporting Information

## Footnotes

6 Please note that the JBC is not responsible for the long-term archiving and maintenance of this site or any other third party hosted site.

## Footnotes

5 The abbreviations used are:

TB: tuberculosis
ITC: isothermal titration calorimetry
NCS: noncrystallographic symmetry
ASU: asymmetric unit
PDB: Protein Data Bank
RMSD: root mean square deviation
DAHP synthase: 3-deoxy-d-arabino-heptulosonate-7-phosphate synthase
CM: chorismate mutase
IPTG: isopropyl 1-thio-β-d-galactopyranoside
Ni-NTA: nickel-nitrilotriacetic acid
BisTris: 2-[bis(2-hydroxyethyl)amino]-2-(hydroxymethyl)propane-1,3-diol
MR: molecular replacement.
