# Supplementary material for: Crystal structure of the TreS:Pep2 complex, initiating α-glucan synthesis in the GlgE pathway of mycobacteria
Source: J Biol Chem. 2019 Mar 15;294(18):7348–59. doi: 10.1074/jbc.RA118.004297 (PMC6509496; doi:10.1074/jbc.RA118.004297)
Supplement: Supporting Information [file supp_294_18_7348__index.html]

Crystal structure of the TreS-Pep2 complex, initiating α-glucan synthesis in the GlgE pathway of mycobacteria — Crystal structure of the TreS–Pep2 complex — Crystal structure of the TreS:Pep2 complex, initiating α-glucan synthesis in the GlgE pathway of mycobacteria — Crystal structure of the TreS:Pep2 complex — Supporting Information 

# Crystal structure of the TreS:Pep2 complex, initiating α-glucan synthesis in the GlgE pathway of mycobacteria

## Supporting Information

- Supporting Information (to be published online) - This file contains Supporting Tables S1 - S3 and Supporting Figures S1 - S10.
