# Supplementary material for: Crystal structure of the TreS:Pep2 complex, initiating α-glucan synthesis in the GlgE pathway of mycobacteria
Source: J Biol Chem. 2019 Mar 15;294(18):7348–59. doi: 10.1074/jbc.RA118.004297 (PMC6509496; doi:10.1074/jbc.RA118.004297)
Supplement: Supporting Information [file supp_RA118.004297_138607_2_supp_301171_pdf3lt.pdf]

Crystal structure of the TreS-Pep2 complex, initiating α-glucan synthesis in the GlgE pathway of mycobacteria

Ali A. Kermani, Rana Roy, Chai Gopalasingam, Klaudia I. Kocurek, Trushar R. Patel, Luke J. Alderwick, Gurdyal S. Besra*, Klaus Fütterer*

From the School of Biosciences, University of Birmingham, Edgbaston, Birmingham, B15 2TT, UK

Running title: *Crystal structure of the TreS–Pep2 complex*

Supporting Information

Supporting Table S1. **X-ray diffraction data and refinement statistics**

**X-ray diffraction data**	
Crystal	TreS–Pep2
Beamline	ID29, ESRF
Wavelength (Å)	0.97717
Space group	$P4_3$
Cell parameters (Å)	315.68, 315.68, 124.95
Complexes per asymmetric unit	2
Resolution range (Å)	49.91 - 3.60
High resolution shell (Å)	3.66 - 3.60
Rmerge (%)[1)]	17.0 (154.2)
Total observations, unique reflections	1090211, 142362
I/σ(I)[1)]	9.1 (1.1)
Completeness (%)[1)]	99.8 (98.1)
Multiplicity[1)]	7.7 (7.0)
$CC_{1/2}$[1), 2)]	0.997 (0.343)
**Refinement**	
Resolution range	49.9 - 3.6
Unique reflections	135356
R_{cryst}, R_{free} (%)	26.1, 28.1
No of non-H atoms	56072
RMSD bonds (Å)	0.006
RMSD angles (º)	1.0
B-factors	
Wilson (Å^2)	126
Average overall (Å^2)	111
RMSD B-factors (Å^2)	1.36
Ramachandran statistics[3)]	
Favoured regions (%)	94.9
Allowed regions (%)	4.5
Disallowed (%)	0.6
Clash score	4.6
Rotamer outliers (%)	1.1

[1)]Values in parentheses refer to the high resolution shell; [2)]$CC_{1/2}$ designates the correlation between random half-datasets according to (1); [3)] Ramachandran statistics were calculated using Molprobity (2).

Supporting Table S2: Amino acid residues buried in the interface between Pep2 (chain I) and TreS (chains A, B). Analysis of buried solvent accessible surface was done using PISA (3). ASA = accessible surface area (in absence of binding partner) in Å^2, BSA = buried surface area in Å^2. HS = type of interaction if not van der Waals or hydrophobic (H = hydrogen bond, S = salt bridge). The bar graphs indicate % of ASA buried (1 bar = 10%) through complexation. Conserved interactions probed by mutagenesis in *M. tuberculosis* are indicated by matching colours and bold type font.

<table>
<tr><th>Pep2</th><th>HS</th><th>ASA</th><th>BSA</th></tr>
<tr><td>I:GLU 67</td><td></td><td>94.15</td><td>26.23 |||</td></tr>
<tr><td>I:TYR 68</td><td></td><td>95.83</td><td>54.08 ||||||</td></tr>
<tr><td>I:PRO 124</td><td></td><td>74.79</td><td>45.02 |||||||</td></tr>
<tr><td>I:VAL 126</td><td></td><td>67.46</td><td>5.86 |</td></tr>
<tr><td>I:PHE 127</td><td></td><td>98.50</td><td>14.73 ||</td></tr>
<tr><td>I:GLU 140</td><td></td><td>70.15</td><td>3.68 |</td></tr>
<tr><td>I:ALA 198</td><td></td><td>55.65</td><td>5.52 |</td></tr>
<tr><td>I:ASN 199</td><td>H</td><td>110.46</td><td>90.79 |||||||||</td></tr>
<tr><td>I:SER 200</td><td>H</td><td>44.06</td><td>23.02 ||||||</td></tr>
<tr><td>I:ALA 201</td><td></td><td>46.25</td><td>42.14 ||||||||||</td></tr>
<tr><td>I:GLU 202</td><td></td><td>69.38</td><td>1.46 |</td></tr>
<tr><td>I:ASP 205</td><td></td><td>80.37</td><td>21.05 |||</td></tr>
<tr><td>I:MET 206</td><td></td><td>44.21</td><td>33.99 ||||||||</td></tr>
<tr><td>I:THR 209</td><td></td><td>76.49</td><td>60.51 ||||||||</td></tr>
<tr><td>I:SER 210</td><td></td><td>5.63</td><td>1.34 |||</td></tr>
<tr><td>I:ARG 212</td><td></td><td>118.51</td><td>41.82 ||||</td></tr>
<tr><td>I:ASP 213</td><td>HS</td><td>57.54</td><td>48.90 |||||||||</td></tr>
<tr><td>I:ALA 216</td><td>H</td><td>67.18</td><td>28.29 |||||</td></tr>
<tr><td>I:GLU 217</td><td></td><td>100.44</td><td>14.56 ||</td></tr>
<tr><td>I:GLU 223</td><td></td><td>70.54</td><td>2.83 |</td></tr>
<tr><td>I:GLU 224</td><td></td><td>81.35</td><td>30.22 ||||</td></tr>
<tr><td>I:VAL 225</td><td></td><td>21.39</td><td>0.25 |</td></tr>
<tr><td>I:GLY 226</td><td></td><td>83.87</td><td>71.10 |||||||||</td></tr>
<tr><td>I:GLY 227</td><td>H</td><td>29.33</td><td>24.51 |||||||||</td></tr>
<tr><td>I:ASP 228</td><td>H</td><td>47.95</td><td>26.32 ||||||</td></tr>
<tr><td>I:PHE 229</td><td></td><td>9.90</td><td>0.49 |</td></tr>
<tr><td>I:GLY 231</td><td></td><td>31.20</td><td>16.31 ||||||</td></tr>
<tr><td>I:GLU 232</td><td></td><td>30.58</td><td>5.83 ||</td></tr>
<tr><td>I:ARG 235</td><td>HS</td><td>114.81</td><td>57.52 ||||||</td></tr>
<tr><td>I:ARG 312</td><td></td><td>71.59</td><td>61.21 |||||||||</td></tr>
<tr><td>I:THR 313</td><td></td><td>19.22</td><td>1.72 |</td></tr>
<tr><td>I:PRO 314</td><td></td><td>79.76</td><td>58.98 ||||||||</td></tr>
<tr><td>I:LYS 315</td><td></td><td>113.83</td><td>29.90 |||</td></tr>
</table>

<table>
<tr><th>TreS</th><th>HS</th><th>ASA</th><th>BSA</th></tr>
<tr><td>B:ARG 311</td><td>H</td><td>88.29</td><td>45.44 ||||||</td></tr>
<tr><td>B:ARG 312</td><td>H</td><td>117.59</td><td>78.54 |||||||</td></tr>
<tr><td>B:GLU 313</td><td></td><td>34.37</td><td>29.98 |||||||||</td></tr>
<tr><td>B:SER 314</td><td></td><td>25.75</td><td>8.28 ||||</td></tr>
<tr><td>B:ARG 315</td><td>H</td><td>17.33</td><td>17.33 ||||||||||</td></tr>
<tr><td>B:PHE 316</td><td></td><td>104.46</td><td>102.50 ||||||||||</td></tr>
<tr><td>B:SER 319</td><td>H</td><td>16.94</td><td>13.34 ||||||||</td></tr>
<tr><td>B:GLU 320</td><td></td><td>51.83</td><td>31.06 ||||||</td></tr>
<tr><td>B:ALA 323</td><td></td><td>78.56</td><td>16.54 |||</td></tr>
<tr><td>B:GLN 324</td><td></td><td>112.23</td><td>16.81 ||</td></tr>
<tr><td>B:TYR 356</td><td></td><td>112.99</td><td>59.18 ||||||</td></tr>
<tr><td>B:GLU 360</td><td>HS</td><td>57.66</td><td>33.45 ||||||</td></tr>
<tr><td>B:LYS 363</td><td></td><td>162.92</td><td>6.81 |</td></tr>
<tr><td>B:ASP 364</td><td></td><td>11.57</td><td>1.96 ||</td></tr>
<tr><td>B:PRO 365</td><td></td><td>92.38</td><td>24.60 |||</td></tr>
<tr><td>B:GLU 497</td><td>H</td><td>55.71</td><td>24.33 |||||</td></tr>
<tr><td>B:LEU 498</td><td></td><td>31.42</td><td>7.27 |||</td></tr>
<tr><td>B:GLY 499</td><td></td><td>18.39</td><td>18.39 ||||||||||</td></tr>
<tr><td>B:GLY 500</td><td></td><td>30.70</td><td>13.45 |||||</td></tr>
<tr><td>B:SER 501</td><td></td><td>93.14</td><td>21.78 |||</td></tr>
<tr><td>B:ASN 502</td><td></td><td>22.21</td><td>3.06 ||</td></tr>
<tr><td>B:PRO 503</td><td></td><td>69.64</td><td>66.46 ||||||||||</td></tr>
<tr><td>B:SER 504</td><td></td><td>12.64</td><td>12.03 ||||||||||</td></tr>
<tr><td>B:ARG 534</td><td>HS</td><td>150.29</td><td>60.39 |||||</td></tr>
<tr><td>B:PHE 535</td><td></td><td>103.12</td><td>52.71 ||||||</td></tr>
<tr><td>B:GLN 537</td><td></td><td>33.00</td><td>15.77 |||||</td></tr>
<tr><td>B:ASN 542</td><td></td><td>80.00</td><td>5.75 |</td></tr>
<tr><td>B:GLN 544</td><td></td><td>66.62</td><td>28.36 |||||</td></tr>
<tr><td>B:GLN 545</td><td></td><td>128.40</td><td>3.64 |</td></tr>
<tr><td>B:GLN 565</td><td></td><td>128.57</td><td>51.60 |||||</td></tr>
<tr><td>B:LEU 566</td><td></td><td>146.30</td><td>36.98 |||</td></tr>
<tr><td>B:PRO 567</td><td></td><td>65.06</td><td>11.21 ||</td></tr>
</table>

<table>
<tr><th>Pep2</th><th>HS</th><th>ASA</th><th>BSA</th></tr>
<tr><td>I:ARG 212</td><td>H</td><td>118.51</td><td>60.15 ||||||</td></tr>
<tr><td>I:ALA 216</td><td></td><td>67.18</td><td>9.94 ||</td></tr>
<tr><td>I:GLU 217</td><td></td><td>100.44</td><td>20.08 ||</td></tr>
<tr><td>I:ARG 357</td><td></td><td>51.24</td><td>24.36 |||||</td></tr>
<tr><td>I:ALA 360</td><td></td><td>41.36</td><td>11.51 |||</td></tr>
<tr><td>I:GLY 361</td><td></td><td>14.88</td><td>1.17 |</td></tr>
<tr><td>I:ASP 363</td><td></td><td>90.46</td><td>3.57 |</td></tr>
<tr><td>I:ASP 364</td><td></td><td>129.12</td><td>67.82 ||||||</td></tr>
</table>

<table>
<tr><th>TreS</th><th>HS</th><th>ASA</th><th>BSA</th></tr>
<tr><td>A:SER 470</td><td></td><td>97.14</td><td>19.40 ||</td></tr>
<tr><td>A:ARG 486</td><td></td><td>134.98</td><td>38.76 |||</td></tr>
<tr><td>A:THR 555</td><td></td><td>76.73</td><td>3.00 |</td></tr>
<tr><td>A:GLY 556</td><td>H</td><td>37.88</td><td>15.88 |||||</td></tr>
<tr><td>A:TYR 557</td><td>H</td><td>139.39</td><td>78.46 ||||||</td></tr>
<tr><td>A:VAL 558</td><td></td><td>80.06</td><td>28.18 ||||</td></tr>
<tr><td>A:GLN 581</td><td></td><td>41.40</td><td>1.23 |</td></tr>
</table>

Supporting Table S3. Isothermal titration calorimetry to Tres-Pep2 binding at variable NaCl concentrations. The sample chamber contained 75 μM *M. smegmatis* TreS at the start of the titration, and Pep2 was titrated to TreS up to a molar ratio of about 1.5 of [Pep2]/[TreS]. Variable n = molar ratio of [Pep2]/[TreS] in TreS–Pep2 complex derived from non-linear regression fitting the binding isotherm. T = 298 K.

	**Pep2 (wild-type)**		
**[NaCl] (mM)**	200	300	400
**pH**	6.5	6.5	6.5
**K_a (10^5 M^{-1})**	2.10 ±0.28	2.22 ±0.23	2.10 ±0.21
**K_d (μM)**	4.8	4.5	4.8
**ΔG^o (kJ/mol)**	-30.2	-30.6	-30.5
**ΔH^o (J/mol)**	-78,660	-116,600	-104,800
**$T\Delta S^o$ (J/mol)**	-48,500	-86,031	-74,312
**n**	0.79 ±0.015	0.54 ±0.01	0.58 ±0.01

Supporting figures

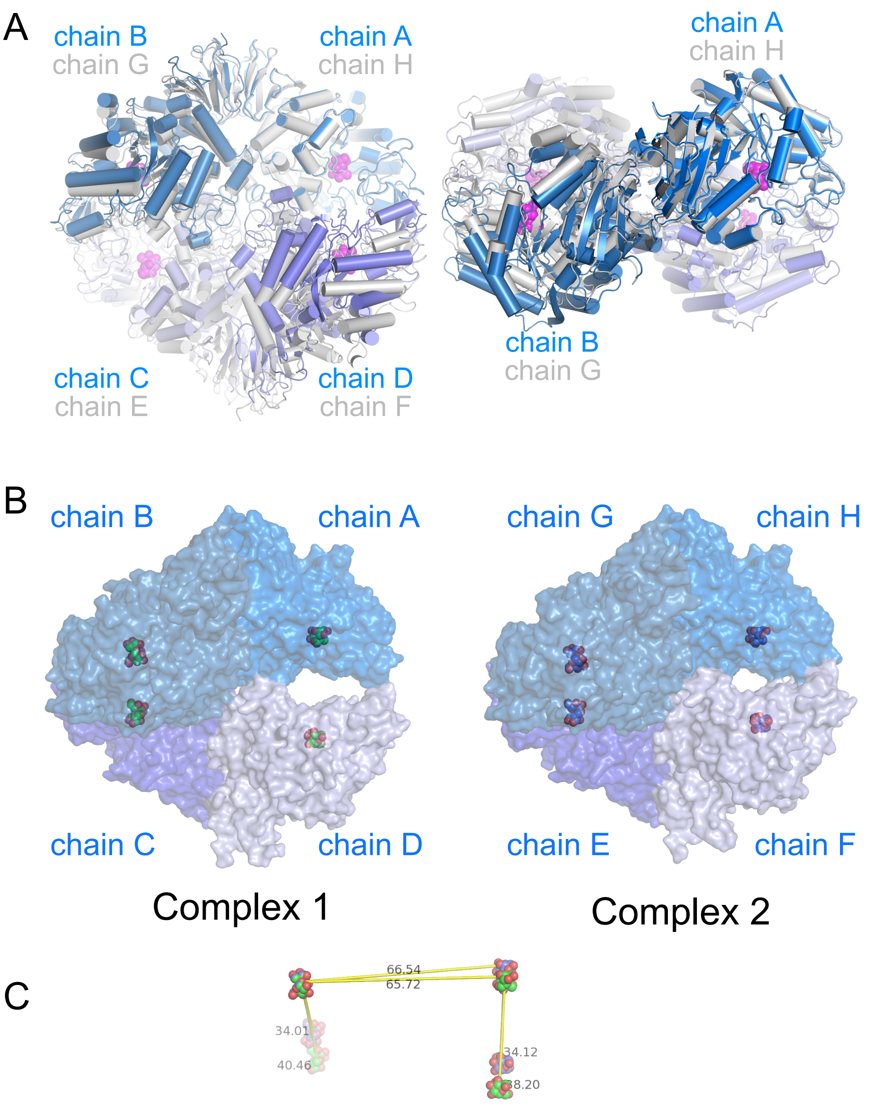

Figure S1. Variation in subunit orientation in TreS tetramer between copies in the asymmetric unit. (A) Superposition of TreS tetramer of complex 1 (chains A – D, blue) over TreS tetramer of complex 2 (chains E – H, grey). Spheres in magenta indicate the location of the active sites. (B) Side-by-side comparison of the molecular surfaces of TreS tetramers of complexes 1 and 2 shown after secondary-structure matched superposition. (C) Distance variation between 'substrate' molecules, resulting from secondary structure-matched superposition of TreS subunits with sucrose-bound *Neisseria polysaccharea* amylosucrase (1ZS2, (4)). The orientations of panels B and C are the same as the orientation shown in panel A. See description in section "*X-ray crystal structure of the* M. smegmatis *TreS–Pep2 complex." of the main paper.*

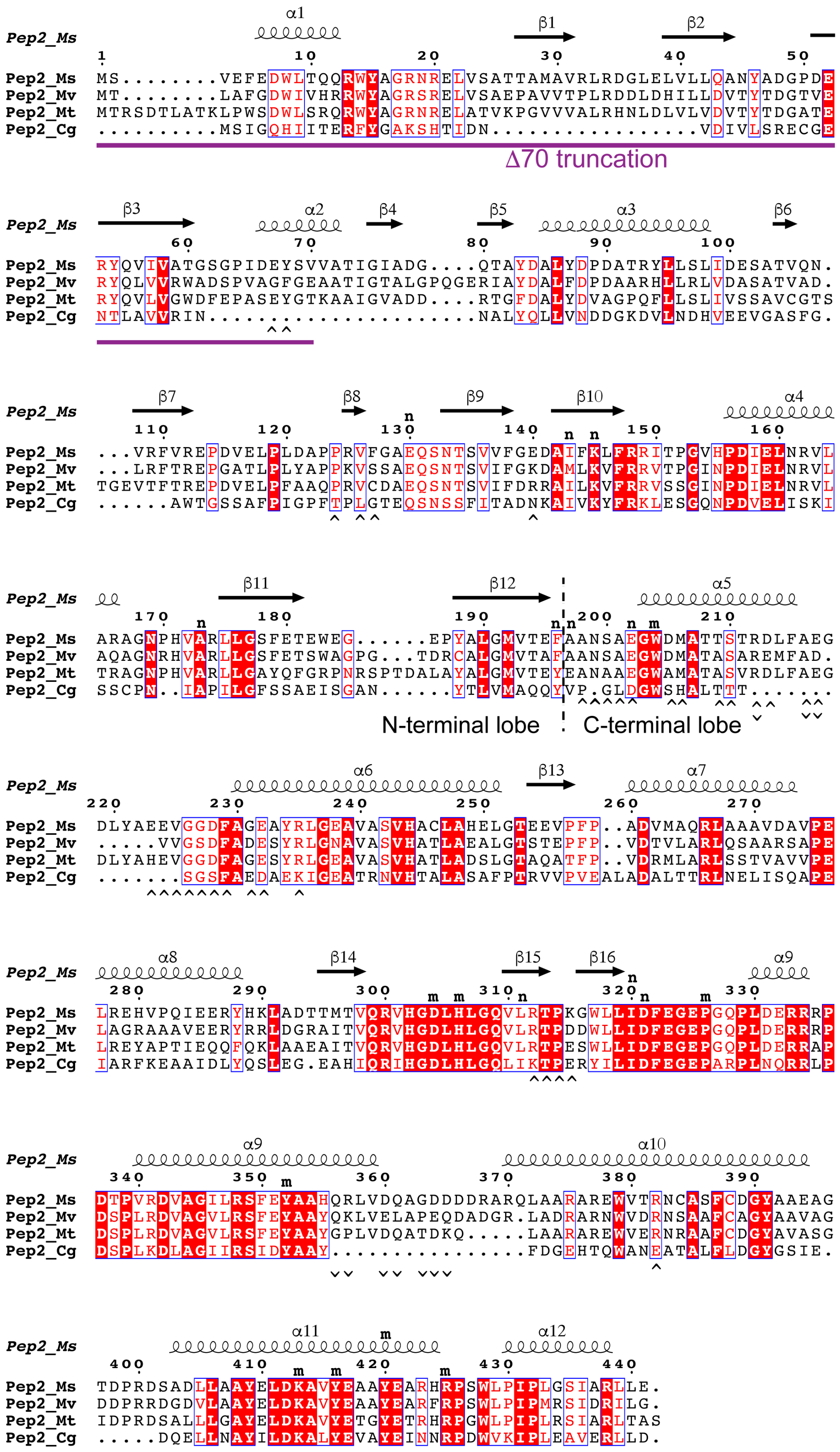

Figure S2. Sequence alignment of Pep2 orthologues and definition of secondary structure elements in the structure of *M. smegmatis* Pep2. The horizontal bar in magenta indicates the extent of the N-terminal truncation in Pep2(Δ70) (70 residues deleted). Upward and downward pointing tick

marks (^, v) indicate Pep2 residues (chain I) in contact with TreS chains B and A, respectively. Lower case letters n, m indicate residues in contact (4 Å distance cut-off) with ATP and maltose, respectively, in the structures of *M. vanbaalenii* (4WZY, (5)) and *M. tuberculosis* Pep2 (4O7P, (6)). The sequence alignment was generated using ClustalX and formatted using Espript (espript.ibcp.fr), whereby boxes indicate sequence similarity (red letters, white background) or identity (white letters, red background), respectively. Species abbreviations are: Ms – *M. smegmatis,* Mv – *M. vanbaalenii*, Mt – *M. tuberculosis*, Cg – *Corynebacterium glutamicum.*

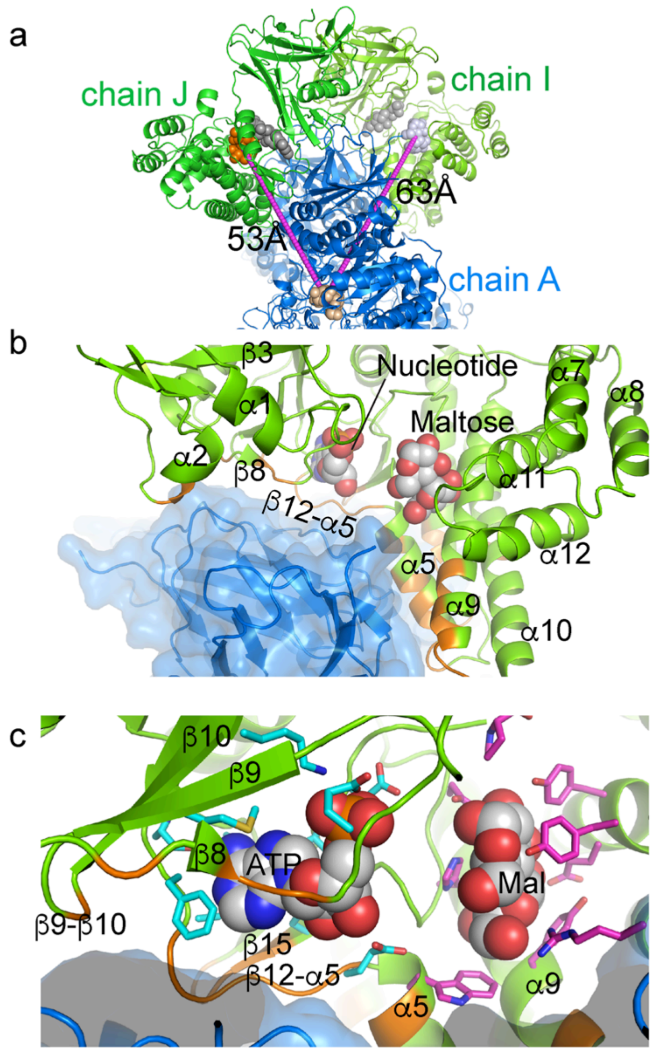

Figure S3. Location of active sites of TreS and Pep2 in the complex. (a) Ribbon diagram of TreS (blue ribbon) and of Pep2 (chains I, J). Spheres indicate position of substrates (as derived from superposition with substrate-bound homologues), lines in magenta the distance between the active site of TreS and two nearest active sites of Pep2, connecting the locations of bound disaccharide. (b, c) Close-up view of the active site of Pep2, with substrates placed according to the structural alignment with ATP-bound *M. vanbaalenii* Pep2 (4WZY, (5)) or maltose-bound *M. tuberculosis* Pep2 (4O7P, (6)). Side chains within 4 Å of the substrates are indicated (cyan – 4WZY, magenta – 4O7P). Structural elements that contribute to the TreS–Pep2 interface (as determined using PISA (3)) are coloured orange and labelled according to Figure S2.

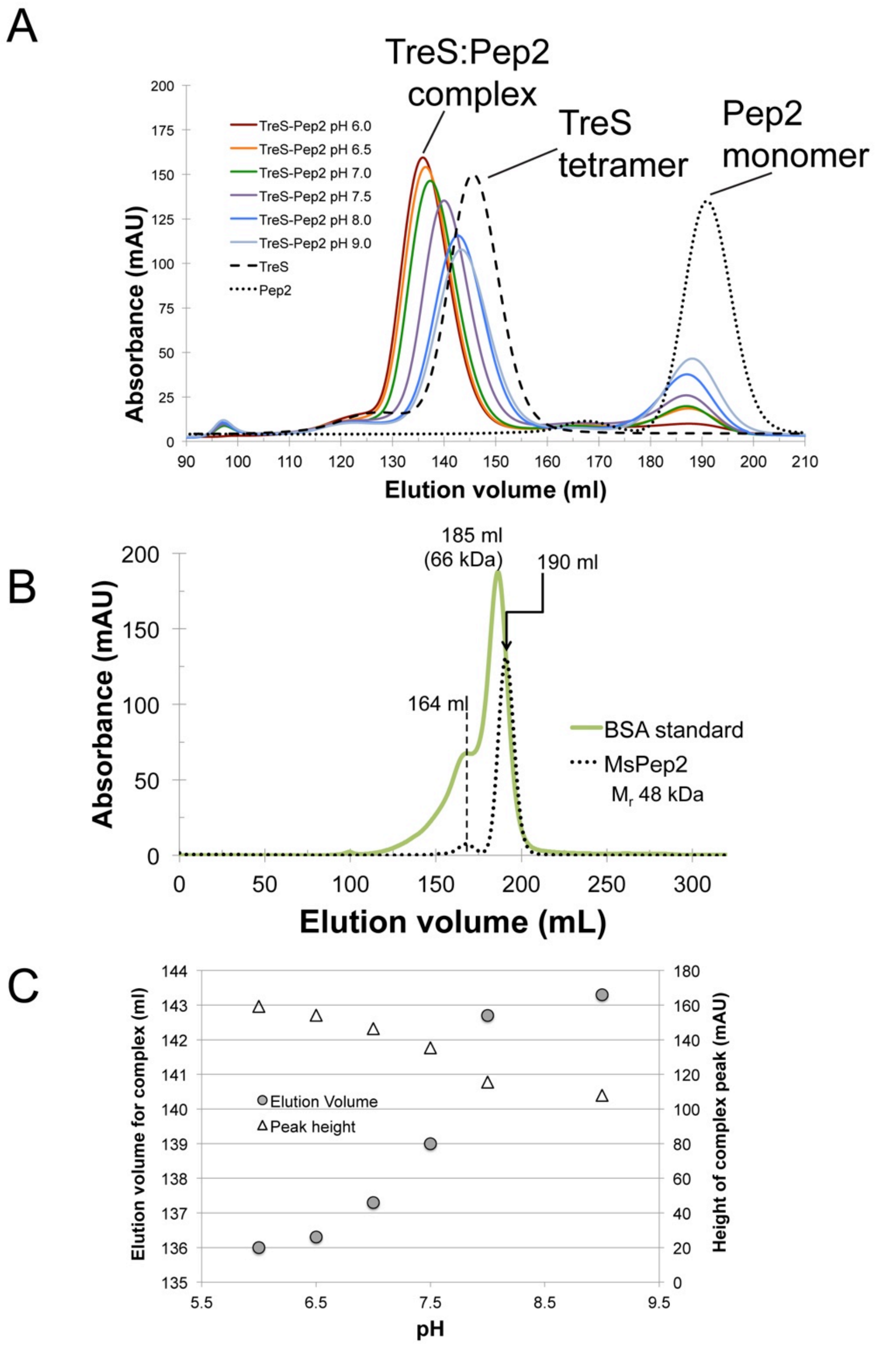

Figure S4. pH-dependent elution profile of the *M. smegmatis* TreS-Pep2 complex from an sephacryl S300-HR size exclusion resin. (**A**) Traces of absorbance (at 280 nm) *vs* elution volume. Proteins were in Na-phosphate buffer at pH values as indicated. (B) Elution of *M. smegmatis* Pep2 in comparison to bovine serum albumin (BSA) standard. The absorbance at the main peak (190 ml) is 127.9 mAU, while the secondary peak (164 ml) is 5.9 mAU, or 4.6% of the height of the main peak. (**C**) Shift of complex peak elution volume (scale on the left) and absorbance of complex peak (scale on the right) as a function of pH.

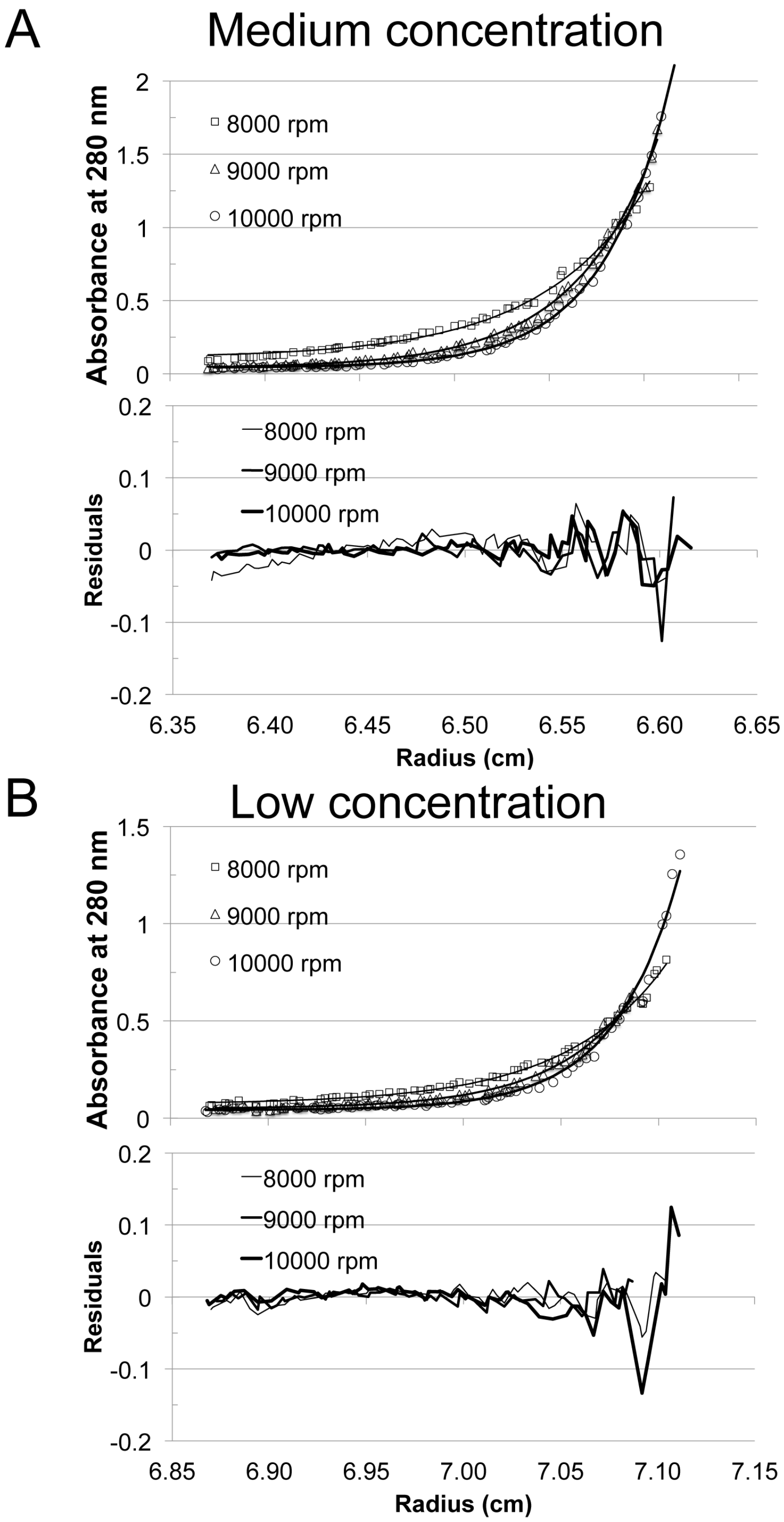

Figure S5. Sedimentation equilibrium analysis of the *M. smegmatis* TreS–Pep2 complex. Sedimentation equilibrium plots (A_{280} *vs* rotor radius) for the medium (2.5 μM, panel a) and low (1.25 μM, panel b) loading concentrations in the 6-window Epon centre piece. Data points are shown as open symbols, and solid lines represent the best fit (top panel) and residuals (lower panel), respectively.

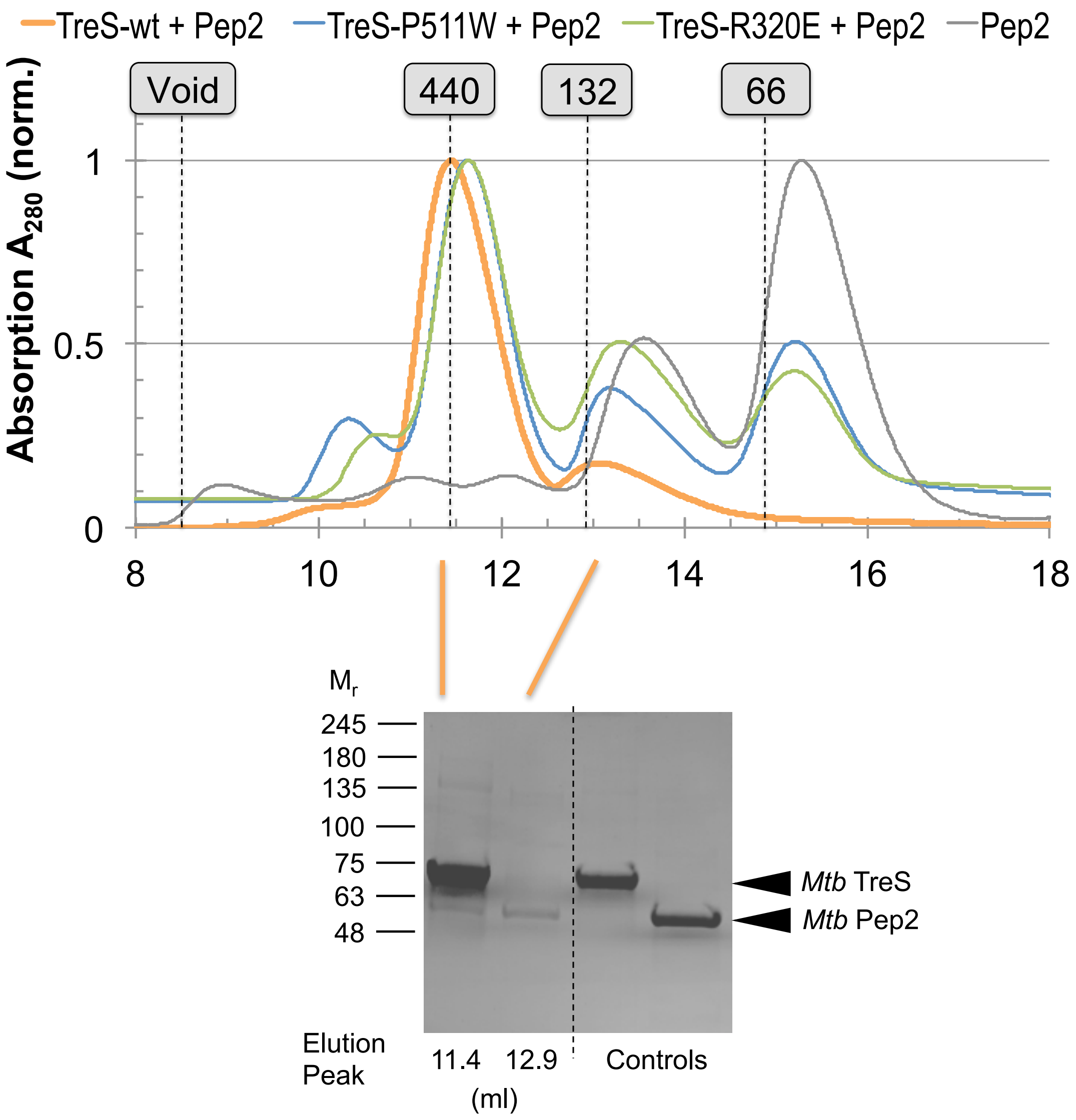

Figure S6. Size exclusion analysis of the *Mtb* TreS–Pep2 complex with mutations at the binding interface. Elution from a Superdex S200 resin (24 ml bedvolume) for *Mtb* TreS (wt), *Mtb* TreS-P511W, *Mtb* TreS-R320E in the presence of an equimolar amount of *Mtb* Pep2. Elution positions of markers ferritin (440 kDa) and of bovine albumin (132 and 66 kDa) are indicated by dashed lines. The bottom panel shows an SDS electrophoresis gel (markers in kDa) of the fractions at 11.4 and 12.9 ml, compared to the purified proteins *Mtb* TreS and *Mtb* Pep2. To illustrate the contrast in elution behaviour for TreS(wildtype)+Pep2 to that of TreS(mutant)+Pep2, this graph shows the elution traces of the TreS+Pep2 runs in Figure 7C, 7D and 7E, respectively, and Pep2 on its own as shown in Figure 7B.

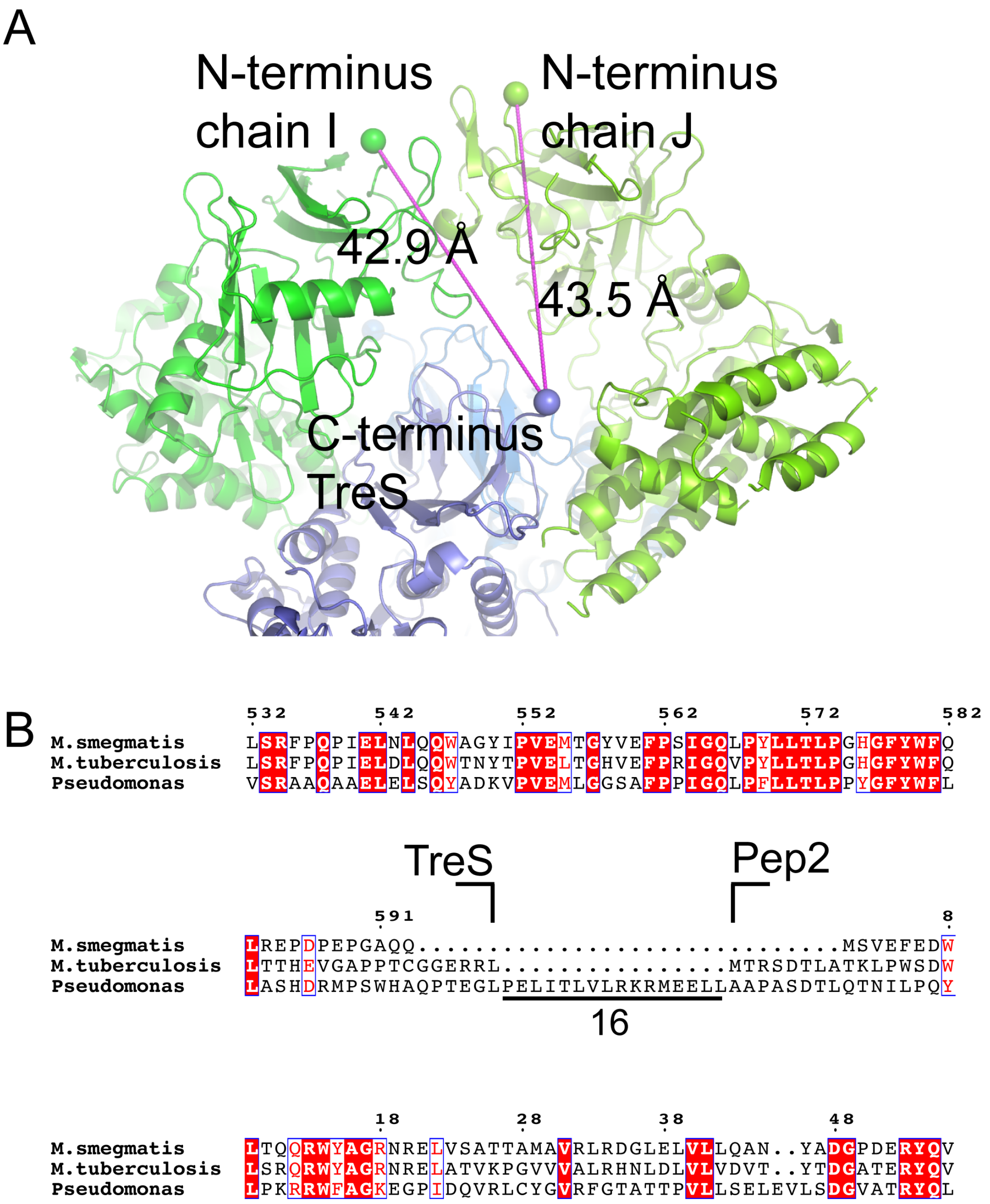

Figure S7. Proximity between C-terminus of TreS and N-terminus of Pep2. (a) Distance measurement between the C-terminus of TreS (chain A, blue sphere) and the first visible residue of Pep2 (green spheres) for chains I and J, respectively. (b) Detail of the sequence alignment of *M. smegmatis* and *M. tuberculosis* TreS/Pep2 with TreS-Pep2 fusion protein of *Pseudomonas aeruginosa*. The physical distance between the termini of TreS and Pep2 is compatible with the length of the linker sequence (underlined residues – 16) in the Pseudomonas fusion protein.

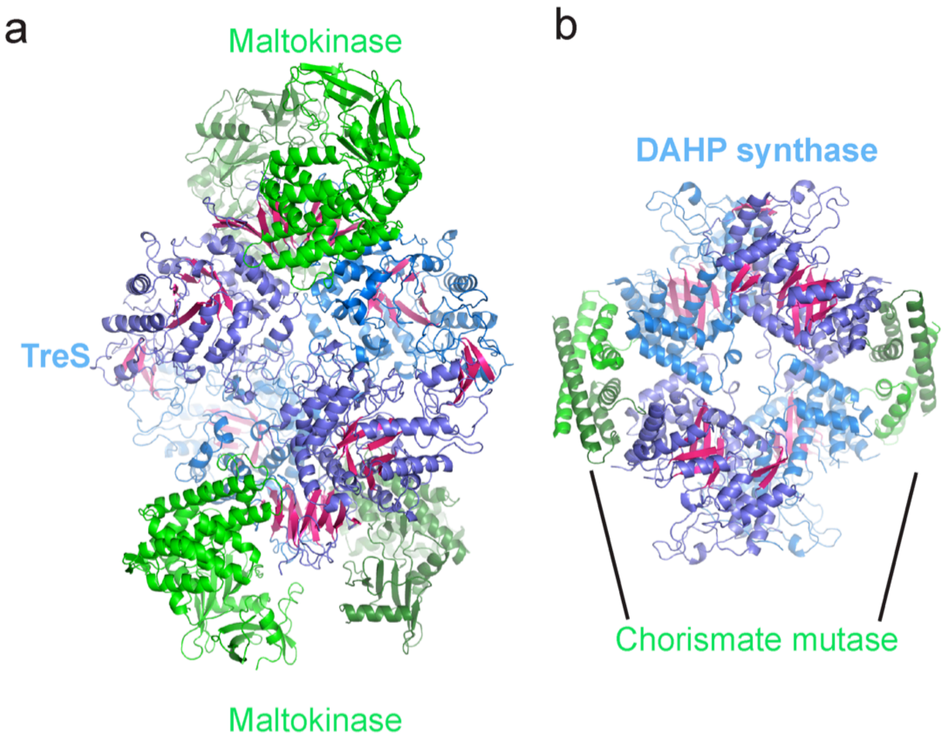

Figure S8. Comparison with the DAHP synthase:chorismate mutase complex and structural rationale for Pep2 rate enhancement. Comparison between the octameric complexes of (a) TreS–Pep2 and (b) *M. tuberculosis* chorismate mutase bound to DAHP synthase (PDB 2W1A, (7)). To highlight the architectural similarity of the two complexes, β-sheet secondary structures are highlighted in magenta.

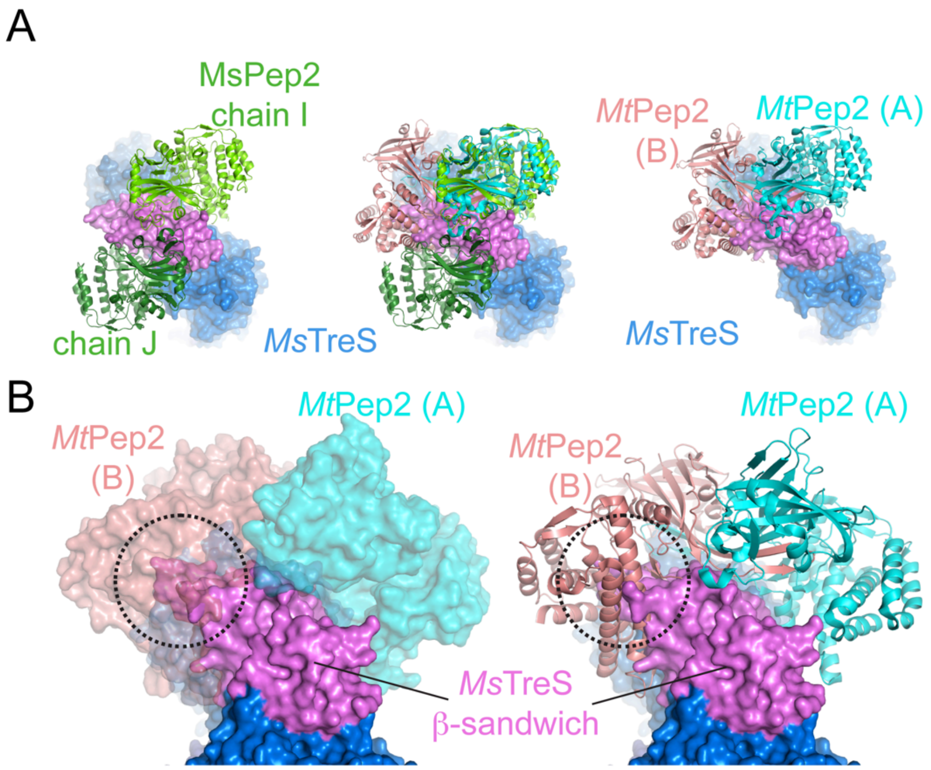

Figure S9. Superposition of the dimer of *M. tuberculosis* Pep2 (*Mt*Pep2) with *M. smegmatis* Pep2 (*Ms*Pep2) in the TreS–Pep2 complex. The *Mt*Pep2 dimer (ribbon in salmon/cyan, PDB entry 4O7P, (6)) was aligned with respect of chain A (cyan) to chain I of *Ms*Pep2 (ribbon in green) in the TreS-Pep2 complex. (a) Illustration of the difference in geometric arrangement of the Pep2 subunits relative to the TreS tetramer, the middle panel representing the merge of the panels on left and right. (b) Illustration of the steric overlap (in the region marked by the dashed circle) between the TreS tetramer (surface in blue and purple) and the non-aligned subunit (chain B, surface/ribbon in salmon) of the MtPep2 dimer.

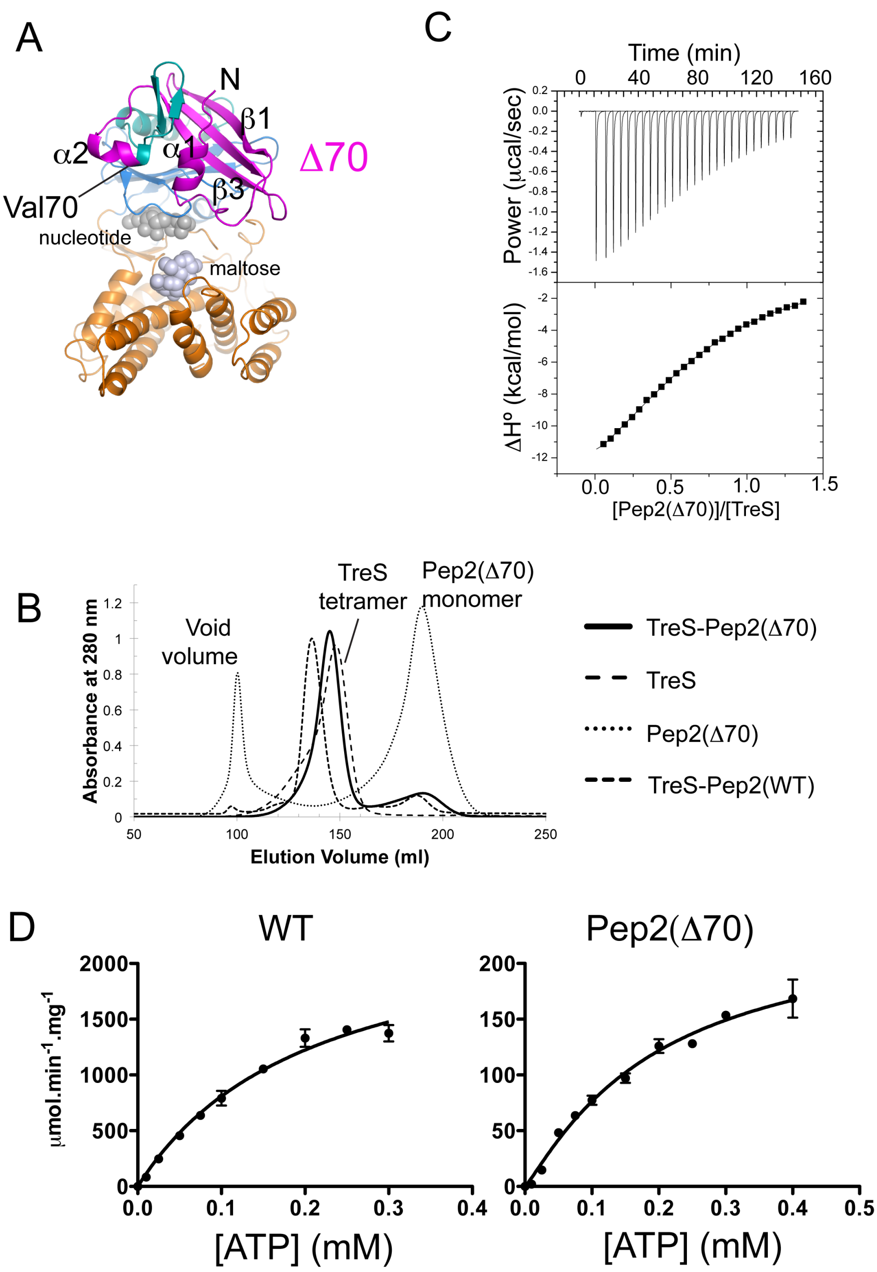

Figure S10. Effects of the N-terminal truncation of 70 residues from the sequence of *M. smegmatis* Pep2. (A) Illustration of the extent of the deleted region (magenta) in the Pep2 monomer, covering the unique β-sheet (strands β1 - β3) in the N-terminal lobe of Pep2. (B) Effect of the Δ70-truncation on association with TreS probed by size exclusion chromatography. The loading concentration for the TreS-Pep2(WT) run was 30 μM (with respect to TreS), while that of TreS-Pep2(Δ70) elution was 75 μM. For ease of comparison, the absorbance values of the TreS-Pep2(WT) elution were scaled to an absorbance of 1 for the complex peak. (C) Probing binding of Pep2(Δ70) to TreS by ITC. The initial concentration of TreS in the reaction chamber was 75 μM. See also Table 1. (D) Effect of the Δ70-truncation on activity of Pep2, varying ATP and holding maltose at a concentration of 100 mM.

References for Supporting Information

1. Karplus, P., and Diederichs, K. (2012) Linking crystallographic model and data quality. *Science* **336**, 1030-1033
2. Davis, I., Murray, L., Richardson, J., and Richardson, D. (2004) MOLPROBITY: structure validation and all-atom contact analysis for nucleic acids and their complexes. *Nucleic Acids Res* **32**, W615-619
3. Krissinel, E., and Henrick, K. (2007) Inference of macromolecular assemblies from crystalline state. *J Mol Biol* **372**, 774-797
4. Skov, L., Mirza, O., Sprogoe, D., van der Veen, B., Remaud-Simeon, M., Albenne, C., Monsan, P., and Gajhede, M. (2006) Crystal structure of the Glu328Gln mutant of Neisseria polysaccharea amylosucrase in complex with sucrose and maltoheptaose. *Biocatal Biotransfor* **24**, 99-105
5. Fraga, J., Maranha, A., Mendes, V., Pereira, P., Empadinhas, N., and Macedo-Ribeiro, S. (2015) Structure of mycobacterial maltokinase, the missing link in the essential GlgE-pathway. *Sci Rep* **5**, 8026
6. Li, J., Guan, X., Shaw, N., Chen, W., Dong, Y., Xu, X., Li, X., and Rao, Z. (2014) Homotypic dimerization of a maltose kinase for molecular scaffolding. *Sci Rep* **4**, 6418
7. Sasso, S., Okvist, M., Roderer, K., Gamper, M., Codoni, G., Krengel, U., and Kast, P. (2009) Structure and function of a complex between chorismate mutase and DAHP synthase: efficiency boost for the junior partner. *EMBO J* **28**, 2128-2142
